# Soil-transmitted helminth infections in free-ranging non-human primates from Cameroon and Gabon

**DOI:** 10.1186/s13071-021-04855-7

**Published:** 2021-07-05

**Authors:** C. Sirima, C. Bizet, H. Hamou, B. Červená, T. Lemarcis, A. Esteban, M. Peeters, E. Mpoudi Ngole, I. M. Mombo, F. Liégeois, K. J. Petrželková, M. Boussinesq, S. Locatelli

**Affiliations:** 1 Institut de Recherche Pour Le Développement (IRD), UMI 233-TransVIHMI-INSERM U1175–University of Montpellier, Montpellier, France; 2 Institute of Vertebrate Biology, Czech Academy of Sciences, Květná 8, 603 65 Brno, Czech Republic; 3 Department of Pathology and Parasitology, Faculty of Veterinary Medicine, University of Veterinary Sciences Brno, Brno, Czech Republic; 4 Projet Prévention du Sida Au Cameroun (PRESICA) and Virology Laboratory IMPM/IRD, Yaoundé, Cameroon; 5 Centre Interdisciplinaire de Recherches Médicales de Franceville, BP 769, Franceville, Gabon; 6 Present Address: Institut de Recherche Pour Le Développement (IRD), Maladies Infectieuses Et Vecteurs : Écologie, Génétique, Évolution et Contrôle (MIVEGEC), IRD 224-CNRS 5290–University of Montpellier, Montpellier, France; 7 Biology Centre, Institute of Parasitology, Czech Academy of Sciences, Ceske Budejovice, Czech Republic

**Keywords:** Soil-transmitted helminths, Non-human primate, Africa, Zoonosis, Phylogeny, Faeces

## Abstract

**Background:**

Zoonotic diseases are a serious threat to both public health and animal conservation. Most non-human primates (NHP) are facing the threat of forest loss and fragmentation and are increasingly living in closer spatial proximity to humans. Humans are infected with soil-transmitted helminths (STH) at a high prevalence, and bidirectional infection with NHP has been observed. The aim of this study was to determine the prevalence, genetic diversity, distribution and presence of co-infections of STH in free-ranging gorillas, chimpanzees and other NHP species, and to determine the potential role of these NHP as reservoir hosts contributing to the environmental sustenance of zoonotic nematode infections in forested areas of Cameroon and Gabon.

**Methods:**

A total of 315 faecal samples from six species of NHPs were analysed. We performed PCR amplification, sequencing and maximum likelihood analysis of DNA fragments of the internal transcribed spacer 2 (ITS2) nuclear ribosomal DNA to detect the presence and determine the genetic diversity of *Oesophagostomum* spp., *Necator* spp. and *Trichuris* spp., and of targeted DNA fragments of the internal transcribed spacer 1 (ITS1) to detect the presence of *Ascaris* spp.

**Results:**

*Necator* spp. infections were most common in gorillas (35 of 65 individuals), but also present in chimpanzees (100 of 222 individuals) and in one of four samples from greater spot-nosed monkeys. These clustered with previously described type II and III *Necator* spp. Gorillas were also the most infected NHP with *Oesophagostomum* (51/65 individuals), followed by chimpanzees (157/222 individuals), mandrills (8/12 samples) and mangabeys (7/12 samples), with *O. stephanostomum* being the most prevalent species. *Oesophagostomum bifurcum* was detected in chimpanzees and a red-capped mangabey, and a non-classified *Oesophagostomum* species was detected in a mandrill and a red-capped mangabey. In addition, *Ternidens deminutus* was detected in samples from one chimpanzee and three greater spot-nosed monkeys. A significant relative overabundance of co-infections with *Necator* and *Oesophagostomum* was observed in chimpanzees and gorillas. *Trichuris* sp. was detected at low prevalence in a gorilla, a chimpanzee and a greater spot-nosed monkey. No *Ascaris* was observed in any of the samples analysed.

**Conclusions:**

Our results on STH prevalence and genetic diversity in NHP from Cameroon and Gabon corroborate those obtained from other wild NHP populations in other African countries. Future research should focus on better identifying, at a molecular level, the species of *Necator* and *Oesophagostomum* infecting NHP and determining how human populations may be affected by increased proximity resulting from encroachment into sylvatic STH reservoir habitats.

**Graphical Abstract:**

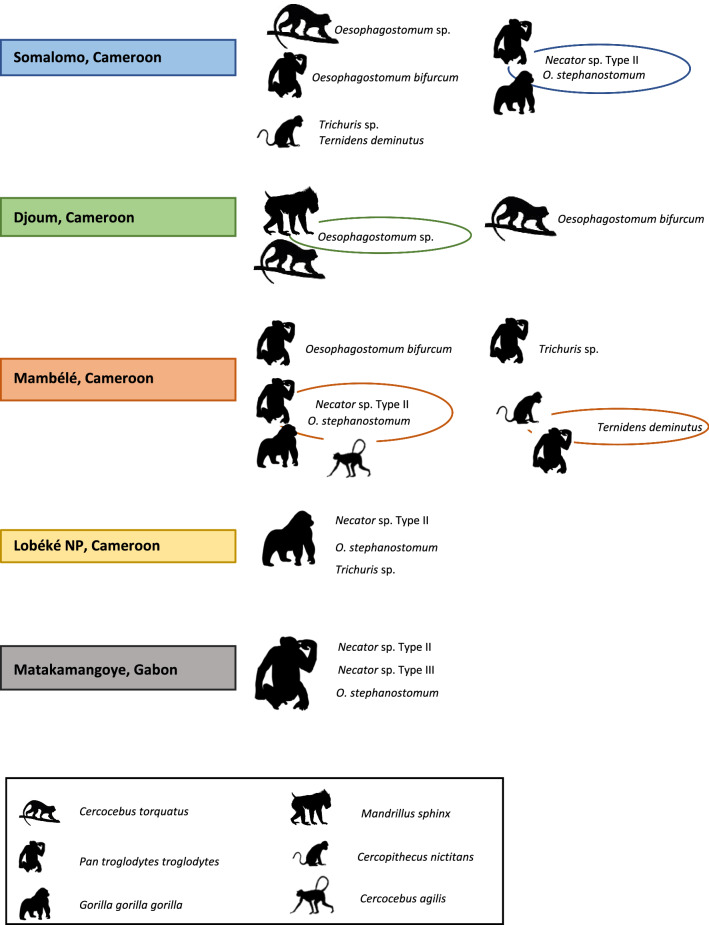

**Supplementary Information:**

The online version contains supplementary material available at 10.1186/s13071-021-04855-7.

## Background

Zoonotic diseases are a serious threat to public health and animal conservation. The risk of zoonotic transmission of viral, bacterial, fungal and parasitic diseases is high between human and non-human primates (NHP) in general, and with the more closely related great apes in particular [1, 2]. Like many NHP, great apes are threatened by forest loss and fragmentation. Unless severely hunted, they are increasingly living in anthropogenically disturbed habitats, such as farmlands, human settlements, fragments of forest and isolated protected areas [3]. The consequence of people and NHP living in increasing spatial proximity to each other [4] is an increasing risk of infection by pathogens contaminating water sources, soil and food [5–10]. Of particular importance among these pathogens are soil-transmitted helminths (STH), which have developmental stages outside of their hosts, allowing for persistence of infective stages in the environment [11]. Infections occur through ingestion or skin penetration of third-stage larvae or eggs. These factors combine to make helminthiases a potential zoonotic risk when humans and NHP share the same habitat.

Approximately 1.5 billion people worldwide are infected with STH (*Ascaris lumbricoides*, *Trichuris trichiura*, *Necator americanus* and *Ancylostoma duodenale*), especially where access to safe drinking water, sanitation and hygiene is poor. The pathology of STH infections in humans includes anaemia, growth retardation and delayed cognitive development [12]. Information on STH prevalence and diversity in African NHP is scarce in some regions, and geographically heterogeneous. Studies results vary according to whether the populations targeted were free-ranging, habituated or (semi-) captive and the analytical approach adopted (morphological or molecular) to identify infections. In NHP, some of these nematodes have unclear clinical significance, while others may have strong pathogenic potential at high intensities. For example, heavy infections of nodular worms (*Oesophagostomum* spp.) are associated with morbidity and mortality in some chimpanzee populations (e.g. in Mahale [13] and Gombe [14], both in Tanzania).

Several cases of bidirectional STH transmission between humans and NHP have been reported [15–18]. *Trichuris* spp. are common in many species of free-ranging NHP in Africa [19, 20] and cryptic lineages, some of which infect also humans, have been detected [16, 21]. Similarly, *Ascaris* has also been found in NHP [22]. Free-ranging chimpanzees [23] and olive baboons [24] can become infected with human roundworms, suggesting that these species are hosts of *Ascari lumbricoides* or a very similar parasite. Gorillas, chimpanzees and several Old World monkey species have been reported to be hosts of *Nectar americanus* based on phenetic characteristics or molecular evidence [25]. Three other *Necator* species (*N. congolensis*, *N. gorillae* and *N. exilidens*) have also been described in African great apes, and *N. gorillae* has been reported to infect humans [17, 26, 27]. Finally, eight species of *Oesophagostomum* have been recorded in free-ranging NHP from the subfamilies *Cercopithecinae* and *Colobinae*, and in chimpanzees (*Pan troglodytes*) [15, 28–30], with three of these (*O. bifurcum, O. oesophagostomum, O. aculeatum*) infecting humans.

The prevalence rate and intensity of STH infections in NHP can vary according to season, social behaviour, degree of arboreality, which affects contact with infectious stages in the soil, and age and sex of the individuals. Sex differences in parasite infections can be the result of differences in home range, foraging dynamics and risk-taking behaviour [31]. The sexes could also differ in their susceptibility to infection because of sex hormones, such as testosterone and oestrogens, which are known to affect immune system function [32].

The World Health Organisation goal for 2030 is morbidity control, defined as reaching < 2% prevalence of medium-to-high intensity infections in preschool-age children and school-age children [12, 33]. In STH endemic countries, re-infection has been observed in half of the children treated for intestinal worms [34], a phenomenon attributed to re-infection due to persistence of infective worm stages in the environment. In addition, the extent to which NHP may act as reservoirs of infection may be affected by landscape perturbations, which facilitate novel ecological associations, new contact zones and host-switching: a pressing problem in forested areas increasingly subjected to anthropogenic disturbance and habitat fragmentation. Therefore, studying STH parasitism in NHP, including African apes, has important public health implications.

In order to formulate effective public health control measures, a better understanding of nematode infections in NHP is needed so that the risk of zoonotic transmission and the potential role of NHP as reservoirs of infections can be determined. This includes monitoring parasite infections, exploring the transmission dynamics among NHP populations and characterising the genetic diversity of the parasites circulating in NHP and human populations.

Thus, the aims of the present study were: (i) to explore the prevalence and genetic diversity of selected genera of STH (*Trichuris*, *Ascaris*, *Necator* and *Oesophagostomum*) infecting gorillas and chimpanzees living in forests of Cameroon and Gabon, and determine the occurrence of these parasites in other NHP species from Cameroon; (ii) to investigate whether the prevalence of STH infection varied among gorillas and chimpanzees across the study sites and whether any co-infection with multiple parasites is observed; and (iii) to examine the presence of parasite sharing among all NHP species investigated, whether parasite occurrence varies with host sex and the potential for zoonotic transmission.

## Methods

### Study sites and faecal collection methods

**Fig. 1:**
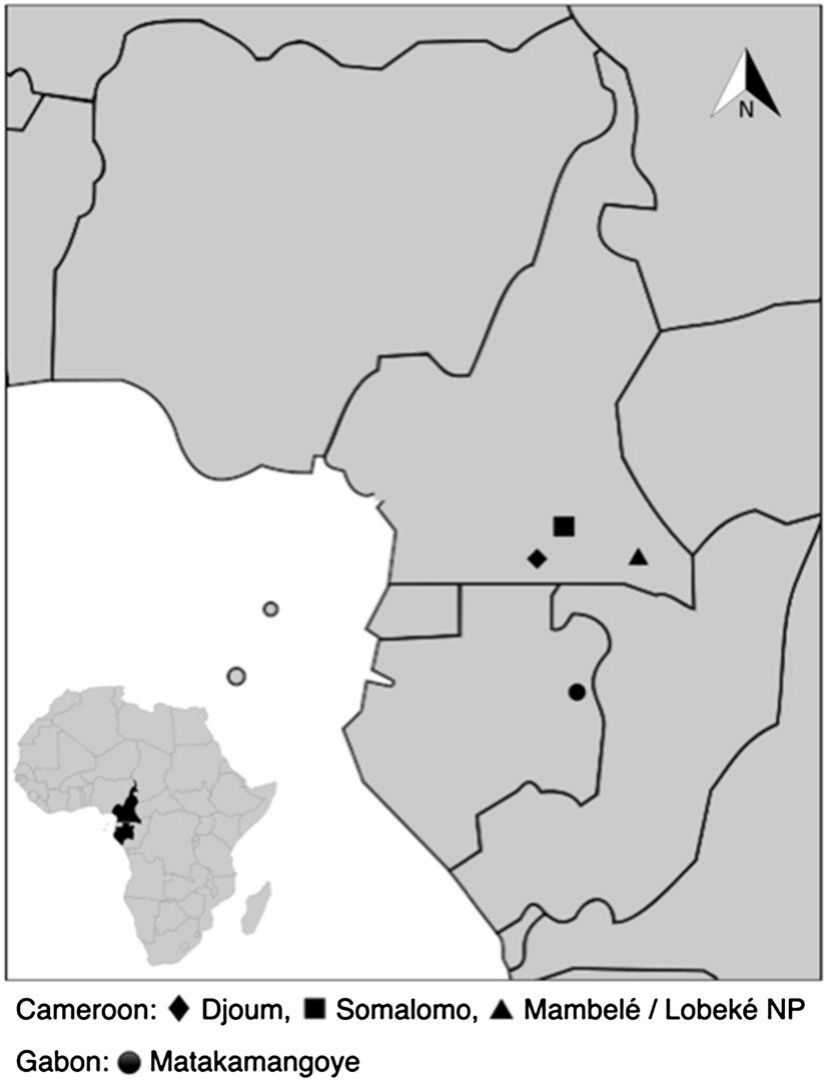
Faecal sample collection sites in Cameroon and Gabon. In Cameroon, the collection sites of Djoum (DJ), Somalomo (SOM) and Mambelé (MB)/Lobeké (LB) National Park are indicated by a filled diamond, filled square and filled triangle, respectively. In Gabon, the collection site of Matakamangoye (MKT) is indicated by a filled circle

To explore to which extent NHP may act as reservoirs of STH infections, we analysed a total of 315 faecal samples from free-ranging, unhabituated NHPs. These samples were fresh when collected (< 1 day old) and were collected non-invasively around NHP nests and feeding places or on traces at four study sites in Cameroon [the forests surrounding the villages of Djoum (DJ) (2°40′00″N, 12°40′00″E); Mambélé (MB) (2°25′00″N, 15°24′00″E); and the neighbouring Lobéké National Park (LB NP) and village of Somalomo (SOM) (3°23′00″N, 12°44′00″E)] (collections between 2008 and 2010), and in primary and secondary forests surrounding the village of Matakamangoye (MKT) (0°6′30″ S, 13°41′57″E) in Gabon (collections between 2009 and 2013) (Fig. 1). These samples had been characterised and analysed in previous studies to determine the prevalence and genetic diversity of Simian Immunodeficiency Viruses (SIV) [35, 36] as well as of filariae [37]. Experienced trackers and/or the researchers identified most faecal samples to be of chimpanzee and gorilla origin and only a few to be of *Cercopithecidae* monkey origin. A 15- to 20-g sample was then placed into a 50-ml tube and mixed with an equal amount of RNA*later*® (Ambion, Austin, TX, USA). The date, time and location [longitude and latitude provided by Global Positioning System (GPS)] of sample collection were recorded along with the collector’s name. Faecal samples were generally kept at ambient temperature for no longer than 2 weeks and subsequently stored at − 20 °C once back in Yaoundé, Cameroon, or Franceville, Gabon. Samples were shipped to Montpellier, France, at ambient temperature and then stored at − 80 °C upon arrival. All samples were transported to France in full compliance with export and import regulations.

### Extractionn of host and parasite DNA from NHP faecal samples

Non-human primate DNA was extracted from faeces using the QIAamp Stool DNA Mini kits (Qiagen, Valencia, CA, USA) following the manufacturer’s instructions. Briefly, 1.5 ml of faecal RNA*later*® mixture was re-suspended in stool lysis buffer and the mixture centrifuged. The supernatants were treated with an InhibitEx tablet, subjected to proteinase K digestion and passed through a DNA binding column. Bound DNA was eluted in 100 μl of elution buffer. To investigate the presence and genetic diversity of STH in NHP faecal samples, a second DNA extraction was performed. A modified protocol was used after the initial resuspension of the sample in the stool lysis buffer. The modification consisted of an additional homogenisation in tubes containing silica beads of different size (lysis matrix E) in a FastPrep-24 mill (MP Biomedical, Eschwege, Germany) and an overnight incubation at 56 °C to improve egg shell lysis and better exposure of the parasite DNA.

### Host species confirmation and identification of individual genotypes by microsatellite analyses

To identify the NHP species infected by the targeted STH, host species confirmation was performed by PCR amplification and sequence analysis of a 460- to 500-bp mitochondrial DNA (mtDNA) fragment spanning the 12S rDNA region, using methods described in previous studies [38]. To determine the number of individuals sampled, the faeces collected from gorillas and chimpanzees were subjected to microsatellite analyses, as previously described [36, 39, 40]. Samples were genotyped at seven loci in two multiplex PCRs (amplifying D18s536, D4s243, D10s676, and D9s922 or D2s1326, D2s1333 and D4s1627). To determine the host sex, a region of the amelogenin gene that contains a deletion in the X but not the Y chromosome was amplified using a *Taq* DNA polymerase core kit (MP Biomedical, Irvine, CA, USA) with 2–10 µl faecal DNA. Samples from the other NHP were subjected to mtDNA analyses but not to microsatellite analyses: to minimise the risk of analysing more than one sample belonging to the same individual, we selected samples collected the same day but in distant, non-overlapping monkey territories. Despite these precautions, and given the low number of samples collected, we will consider them as samples and not as separate individuals.

### PCR screening of soil-transmitted helminths

To better understand which NHP species are mostly infected with the STH, therefore representing the most prevalent reservoir, semi-nested PCR assays targeting the internal transcribed spacer 2 (ITS2) marker were used to determine the presence of *Trichuris* spp., *Oesophagostomum* spp. and *Necator* spp. in all 315 samples [15, 41, 42]. To detect the presence of *Ascaris* spp., we performed a nested PCR targeting the ITS1 marker [43]. Primer details, PCR conditions and expected amplicons size are given in Table 1. All PCR reactions were performed in a 50-μl reaction volume containing 10 and 5 µl of template DNA for primary and semi-nested/nested PCR, respectively, 10 pM of each primer, 25 µl HotStarTaq Master Mix (Qiagen, Hilden, Germany), providing a final concentration of 1.5 mM MgCl_2_ and 200 μM of each dNTP. For the first round of PCR, bovine serum albumin (Sigma-Aldrich, St. Louis, MO, USA) was added at a final concentration of 0.2 μg/ml to improve amplification success. The reactions were performed on a Bio-Rad T100™ thermal cycler (Bio-Rad, Hercules, CA, USA). The amplicons were electrophoresed on 1% agarose gels stained with ethidium bromide. After purification (in 2% agarose gel) with the GeneClean® Turbo Kit (Qbiogene, Inc., Carlsbad, CA, USA), the PCR products were sequenced with the primers of the second step (if nested or semi-nested) on an automated sequencer (3500 Genetic Analyzer; Applied Biosystems, Foster City, CA, USA), and the resulting sequences were analysed using SeqMan DNASTAR software (Lasergene; DNASTAR, Inc., Madison, WI, USA). At least two attempts were made to obtain high-quality sequences by adapting the quantity of PCR-purified DNA used in the sequencing reactions. Low-quality sequences (short amplicon size and/or sequences displaying background noise with too many unresolved degenerated nucleotides) were excluded from further phylogenetic analyses (details on the number of sequences obtained that were of satisfactory size and quality are given in Table 2).

**Table 1:** Primers and PCR cycling programmes

Target organism	Gene	Primer	Sequence (5'–3')	Product size (bp)	Thermal profile^a^						
					Step 1		Step 2		Step3		*N*
					Temperature (°C)	Duration (s)	Temperature (°C)	Duration (s)	Temperature (°C)	Duration (s)	
*Oesophagostomum*/*Necator*	*ITS2*	NC1	ACGTCTGGTTCAGGGTTGTT	NA	94	30	50	30	72	45	45
		NC2	TTAGTTTCTTTTCCTCCGCT								
		OesophITS2	TGTRACACTGTTTGTC-GAAC	250/300	94	30	55	30	72	30	35
		NC2	TTAGTTTCTTTTCCTCCGCT								
*Trichuris trichiura*	*ITS2*	ExtITS2	GGATCACTTGGCTGGTAG	NA	94	30	56	30	72	45	45
		NC2	TTAGTTTCTTTTCCTCCGCT								
		IntITS2	CTTGAATACTTTGAACGCACATTG	480–700	94	30	49	30	72	45	35
		NC2	TTAGTTTCTTTTCCTCCGCT								
*Ascaris lumbricoides*	*ITS1*	ITS F1	CGAGCAGAAAAAAAAAAGTCTCC	NA	94	30	50	45	72	45	45
		ITS R1	GGAATGAACCCGATGGCGCAAT								
		ITS F2	CGAGCAGAAAAAAAAAAAAGTCTCC	~ 500	94	30	52	30	72	30	35
		ITS R2	GCTGCGTTCTTCATCGAT								

* ITS* Internal transcribed spacer, *N* number of cycles,* NA* data not available

All PCR analyses started with 95 °C for 15 min and finished with 72 °C for 10 min

^a^Step 1: denaturation; Step 2: annealing; Step 3: elongation

**Table 2:** Summary of results of PCR-positive individuals/samples with *ITS2* sequences exploitable for phylogenetic analyses

Locality	NHP species	*n*^a^	PCR-positive for *Necator* spp.	*ITS2* phylogeny *Necator* spp.	PCR-positive for *Oesophag.*sp.	*ITS2 * phylogeny* Oesophagostomum*. spp.	PCR-positive for *Trichuris* spp.	*ITS2 * phylogeny* Trichuris* spp.
Djoum (CAM)	Mandrill	12	0	–	8	3	0	–
	Red-capped mangabey	7	0	–	3	2	0	–
	Greater spot-nosed monkey	1	0	–	0	–	0	–
Somalomo (CAM)	Chimpanzee	23	20 (87.0%)	9	22 (95.7%)	17	0	–
	Gorilla	38	24 (63.2%)	1	38 (100%)	26	0	–
	Red-capped mangabey	2	2	0	2	1	0	-
	Greater spot-nosed monkey	2	1	0	2^b^	2^b^	1	1
Mambélé (CAM)	Chimpanzee	59	17 (28.8%)	10	34 (+ 1^b^) (57.6%)	22 (+ 1^b^)	2 (3.4%)	1
	Gorilla	12	2 (16.7%)	1	8 (66.7%)	8	1 (3.7%)	1
	Agile mangabey	3	1	1	2	2	0	–
	Greater spot-nosed monkey	1	0	-	1^b^	1^b^	0	–
Lobéké NP (CAM)	Gorilla	15	9 (60.0%)	2	5 (33.3%)	1	0	-
Matakamangoye (GAB)	Chimpanzee	140	63 (45.0%)	50	101 (72.1%)	90		
Total		315	139(44.1%)	75	223 (+ 4^b^) (70.8%)	172 (+ 4^b^)	4 (1.3%)	3

CAM Cameroon, GAB Gabon, *NHP* non-human primates,* NP* National Park

^a^*n* represents the number of individuals tested (chimpanzee, gorilla) or of samples tested (all other NHP species)

^b^Sequences of *Ternidens deminutus* not used in the phylogenetic tree

### Molecular identification and phylogenetic analysis

To explore the genetic diversity of the nematodes present in the NHP studied and to evaluate their zoonotic potential, we first confirmed the identity of these nematodes, searching through nucleotide BLAST [44] (https://blast.ncbi.nlm.nih.gov/Blast.cgi). All sequences were aligned in Geneious Prime 2020.1.2 (https://www.geneious.com) guided by CLUSTALW implemented in Geneious. The sequences of *Oesophagostomum* containing ambiguous nucleotides were submitted to haplotype reconstruction using “Open Unphase/Genotype data” in DnaSP 6 [45]. The reconstructed haplotypes were used in further analyses. Pairwise sequence distances were calculated in Geneious. Sequences representing different haplotypes and/or haplogroups were subsequently used in the phylogenetic analyses. GenBank sequences of the species found in humans and NHP were narrowed down to haplogroups. Each haplogroup was represented by one sequence in the final alignment. A maximum likelihood (ML) analysis was carried out in IQ-TREE 1.6.11 [46]. The most suitable model was chosen by ModelFinder [47] implemented in IQ-TREE based on the highest Bayesian information criterion scores and weights (BIC). The tree topology was tested by 1000 replicates of ultrafast bootstrap [48] and the Shimodaira–Hasegawa (SH)-like approximate likelihood ratio test [49].

### Statistical analysis

To explore the STH co-infection patterns in the NHP species studied, we investigated whether *Oesophagostomum* and *Necator* occurred together (i.e. in the same faecal samples) more or less frequently than expected by chance. We ran a two-tailed Fisher’s exact test for chimpanzees and gorillas separately, and for these two species pooled together. We excluded mandrills, mangabeys and *Cercopithecus* monkeys from this analysis due to the low number of samples collected. Using the same test, we compared the prevalence of infection with *Oesophagostomum* or *Necator* between male and female chimpanzees in Gabon (the only site where we had complete results for the amelogenine microsatellite analysis).

## Results

### Host species and individuals’ genotypes

The 315 samples analysed in our study originated from six species: 65 from gorilla (*Gorilla gorilla gorilla*), 222 from chimpanzee (*Pan troglodytes troglodytes*), 12 from mandrill (*Mandrillus sphinx*), nine from red-capped mangabey (*Cercocebus torquatus torquatus*), three from agile mangabey (*Cercocebus agilis*) and four from greater spot-nosed monkey (*Cercopithecus nictitans*). Based on the microsatellite analyses, both gorilla and chimpanzee samples corresponded to 65 and 222 different individuals, respectively. Among the 315 samples analysed, we obtained information on the sex of only 114 chimpanzees from Matakamangoye. Amelogenine gene analyses results indicated that 40 chimpanzee samples were from females and 74 from males. As the number of other cercopithecid primate samples was low and the host individuals were not genotyped, no statistical analyses could be performed on these samples. The number of samples collected and the NHP species distribution of these samples across sites is detailed in Table 2.

### Prevalence of STH infections

The prevalence of all analysed STH taxa in gorillas and chimpanzees and the presence of these taxa in mandrills, mangabeys and greater spot-nosed monkeys is summarised in Table 2. *Oesophagostomum* was the most frequently occurring parasite, present in most of the NHP species investigated, with a prevalence of 78.5% (51/65) in gorillas and 70.7% (157/222) in chimpanzees. *Oesophagostomum* DNA was also commonly detected in other NHP host species, with the exception of greater spot-nosed monkeys (Table 2). *Necator* DNA was detected by PCR in roughly half of both gorilla and chimpanzee samples, 53.8% (35/65) and 45.0% (100/222) respectively, and in one of the four greater spot-nosed monkey samples. *Trichuris* was detected very rarely, in just two of the 222 chimpanzees (0.9%), in one of the 65 gorillas (1.5%) and in one of the four greater spot-nosed monkey samples (Table 2). *Ascaris* was not detected in any of the analysed samples. In addition, PCR amplicons from assays targeting *Necator* and *Oesophagostomum* from a chimpanzee and three greater spot-nosed monkey samples were identified by BLAST sequence analyses as *Ternidens deminutus*.

In both gorillas and chimpanzees, the highest prevalence of *Oesophagostomum* and *Necator* was observed in Somalomo, Cameroon, followed by Matakamangoye, Gabon, and the lowest prevalence was observed in animals originating from Mambélé, Cameroon. Only gorilla samples were collected in the Lobéké NP, where the prevalence of *Necator* and *Oesophagostomum* reached 60.0 and 33.3% respectively.

Among the surveyed host species, gorillas were the species most infected by either *Necator*, *Oesophagostomum* or by both taxa (86.2%), followed by chimpanzees (81.5%). Mandrills were positive only for *Oesophagostomum*, greater spot-nosed monkeys were positive only for *Necator*, while both parasites were detected in red-capped and agile mangabeys. *Necator*/*Oesophagostomum* co-infections were detected in 30 gorillas (46.1%), in 76 chimpanzees (34.2%), in two red-capped mangabey samples and in one of three agile mangabey samples (Table 3).

**Table 3:** *Necator* and *Oesophagostomum* PCR-positive individuals (gorilla, chimpanzee)/samples (other NHP)

Host	*n*	PCR-positive *Necator* only	PCR-positive *Oesophagostomum* only	*Necator*/*Oesophagostomum* co-infections	*Necator* OR *Oesophagostomum* OR both parasites
Chimpanzee	222	100 (45%)	157 (70.7%)	76 (34.2%)	181 (81.5%)
Gorilla	65	35 (53.8%)	51 (78.5%)	30 (46.1%)	56 (86.2%)
Mandrill	12	0	8	0	8
Red-capped mangabey	9	2	5	2	5
Agile mangabey	3	1	2	1	2
Greater spot-nosed monkey	4	1	0	0	1
Total	315	139	223	109	253

Values in table are presented as the number (*n*) of animals with the percentage of total given in parentheses where appropriate

### Occurrence of *Oesophagostomum* and *Necator* co-infections in chimpanzees and gorillas

We first analysed chimpanzees and gorillas separately and observed that co-infection with *Oesophagostomum* and *Necator* occurred frequently, but that the association was not statistically significant. When the two NHP species were combined and analysed together, co-infections occurred more often than expected by chance (two-tailed Fisher’s exact test: *p* = 0.023; Additional file 1: Table S1).

### Parasitic infections according to the sex of the individual analysed

Sex was determined for 114 of the 140 chimpanzee samples from Gabon: 40 females and 74 males. The prevalence of *Oesophagostomum* or *Necator* did not differ significantly between males and females (two-tailed Fisher’s exact test: *p* = 0.645 and 0.326, respectively; Additional file 1: Table S2).

### Phylogenetic analyses

A total of 254 sequences of *Necator*, *Oesophagostomum*,* Ternidens deminutus* and/or *Trichuris* was obtained from 204 faecal samples (Table 2). The success rate of obtaining high-quality sequences from the PCR products was 53.9% for *Necator*, 77.1% for *Oesophagostomum* and *Ternidens deminutus* together and 75% for *Trichuris.* Sequences generated in this study that represent unique haplotypes were deposited in GenBank (Accession numbers given in Additional file 2: Table S1-B, S2-B and S3)*.* Table 4 provides an overview of the different parasite species detected in the NHP hosts according to the study site, with haplotypes listed when applicable, and the number of individuals/samples for which sequences were obtained.

**Table 4:** Occurrence of different species of* Necator*,* Oesophagostomum*,* Trichuris* and *Ternidens* according to the NHP sampled and the collection location

Parasite Locality	NHP species^a^	*Necator* type II^b^	*Necator* type III^b^	*O. stephanostomum*^b^	*O. bifurcum*^b^	*Oesophagostomum* spp.^b^	*Trichuris* spp.^b^	*Ternidens deminutus*^b^
Djoum (CAM)	Mandrill *n* = 3	**–**	**–**	–	–	3 OH15	**–**	**–**
	Red-capped Mangabey *n* = 2	**–**	**–**	**–**	1 OH9,12	1 OH13	**–**	**–**
Somalomo (CAM)	Chimpanzee *n* = 17	9 NH2, 1/2, 1/2/4	**–**	14 OH1,2,4,5	3 OH9,10,11	**-**	**–**	**–**
	Gorilla *n* = 26	1 NH2/4	**–**	26 OH1,2,3,5,7,8	**–**	**-**	**–**	**–**
	Red-capped Mangabey *n* = 1	**–**	**–**	**–**	**–**	1 OH13,14	**–**	**–**
	Greater spot-nosed monkey *n* = 2	**–**	**–**	**–**	**–**	**–**	1	2
Mambélé (CAM)	Chimpanzee *n* = 30	10 NH1,2,3,4	**–**	21 OH1,2,3,4,5	1 OH9	**–**	1	1
	Gorilla *n* = 8	1 NH1/2	**–**	8 OH1,2,3	**–**	**–**	**-**	**–**
	Agile Mangabey *n* = 2	1 NH1/2	**–**	2 OH1,3	**–**	**–**	**-**	**–**
	Greater spot-nosed monkey *n* = 1	**-**	**–**	**–**	**–**	**–**	**-**	1
Lobéké NP (CAM)	Gorilla *n* = 4	3 NH1,1/2	**–**	1 OH1	**–**	**–**	1	**–**
Matakamangoye (GAB)	Chimpanzee *n* = 108	49 NH1,2	1 NH5	90 OH1,2,3,4,5,6,16	**–**	**–**	**–**	**–**
Total		74	1	162	5	5	3	4

^a^*n* is the number of individuals (gorilla, chimpanzee) or samples (other NHP) from which exploitable sequences were obtained

^b^Numbers indicate the number of high-quality partial sequences of the ITS2 region (138–273 bp) obtained. Haplotypes are shown where applicable

#### *Necator*

Of 139 *Necator* PCR-positive samples, we obtained 75 high-quality partial sequences of the ITS2 region (138–273 bp). These sequences originated from 19 chimpanzees, five gorillas and one agile mangabey sample from Cameroon, and from 50 chimpanzees from Gabon (Additional file 2: Table S1-B). A total of five different variants (NH1–NH5) of ITS2 was detected; of these (NH1–NH4 differed by three single nucleotide polymorphism (SNP) positions, and haplotype NH5 differed from the others by 3.7–5.6%. Two genotypes (NH3, NH4) had one ambiguous nucleotide “Y” that differed in its position within the alignment. Minimal differences were observed among variants NH1–NH4; therefore, these will be referred to as haplogroup NH1-4. Haplotype NH1 (*n* = 14) was detected in gorillas and chimpanzees from both Gabon and Cameroon (Lobéké and Mambélé), while haplotype NH2, the most frequent haplotype (*n* = 32), was detected only in chimpanzees from both countries. Variants NH3 and NH4 were both represented by a single sequence and both were detected in chimpanzee samples from Mambélé, Cameroon. Haplotype NH5 was found in a single chimpanzee sample from Matakamangoye, Gabon. Unfortunately, 26 sequences were too short to be assigned to one particular haplotype; 23 of these sequences were identical to both NH1 and NH2 (comprising sequences from two gorillas from Mambélé and Lobéké, one agile mangabey sample from Mambélé and 21 chimpanzees from different study sites), and three were identical to NH1, NH2 or NH4 (2 chimps and a gorilla from Somalomo).

**Fig. 2:**
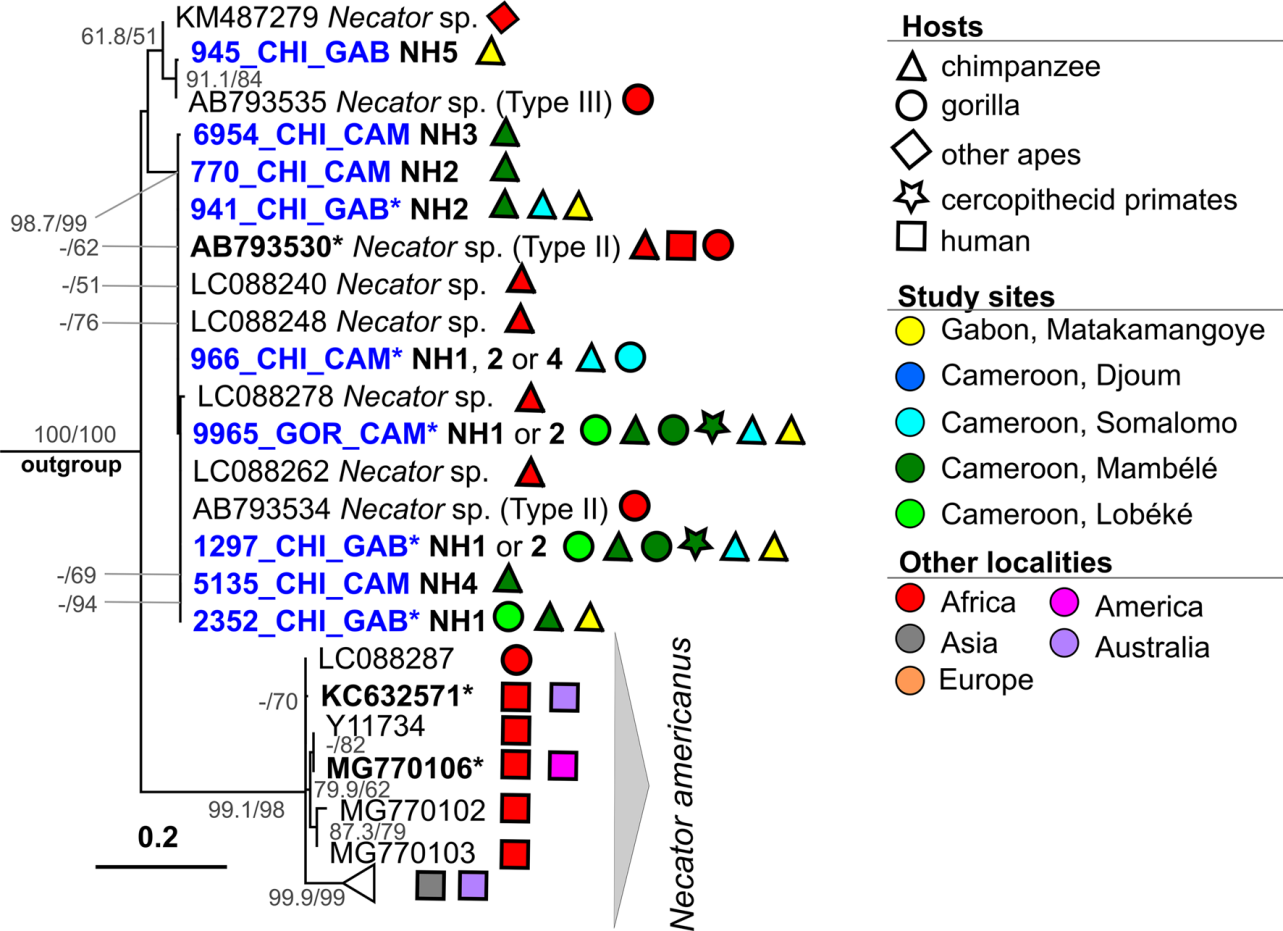
Maximum likelihood phylogenetic tree of the internal transcribed spacer 2 (ITS2) region of *Necator* spp. inferred by HKY + F model. The phylogeny was tested by the SH-aLRT nonparametric test (left) and ultrafast bootstrap (right) in 1000 replicates. Only values > 50 (in %) are shown. Sequences originating from this study are given in blue. Sequences in bold and those marked with an asterisk indicate haplogroups comprising multiple sequences

Nine *Necator* sp. sequences representing all five variants/haplogroups were combined in a final alignment of 310 bp, comprising a total of 40 sequences (31 accessed from GenBank) of *Necator* spp. and two additional sequences of *Bunostomum trigonocephalum* (GenBank accession numbers AY439022, MG182023) added as an outgroup. The complete list of sequences and their assignment to haplogroups can be found in Additional file 2: Tables S1-A and S1-B. The ML phylogenetic tree was computed by the HKY + F model. The resulting tree showed two distinct clades, with the first comprising only *N. americanus* sequences, originating mainly from humans, and the second comprising only sequences labelled as *Necator* sp., originating predominantly from NHP, which were further divided in two sub-clades (Fig. 2). Our haplotype NH5 sequence clustered very closely with a sequence of *Necator* sp. type III (AB793535) published by Hasegawa et al. [17] that originated from a human from the Central African Republic (CAR). All sequences representing the other haplogroup (NH1–NH4) clustered in a different sub-clade, although the node was not strongly supported. This sub-clade also comprised sequences of *Necator* sp. originating from chimpanzees (Uganda), gorillas (Gabon, CAR) and a human from CAR [17, 50]. Hasegawa et al. [17] labelled this taxon as *Necator* sp. type II.

#### *Oesophagostomum*

Of 227 *Oesophagostomum* PCR-positive samples we obtained 176 high-quality partial sequences of the ITS2 region (171–174 bp). These sequences were subsequently unfolded to 214 phased sequences belonging to 17 unique haplotypes, labelled OH1–17. The list of sequences and their assignment to haplotypes is provided in Additional file 2: Table S2-B. Thirteen sequences were too short to be distinguished as either haplotype OH1 or OH2. All haplotypes but one had some *Oesophagostomum* species as the closest match in BLAST. The haplotype OH17 (4 sequences) had a 100% match with a sequence of *Ternidens deminutus* (AJ888729). This haplotype comprised sequences originating from a chimpanzee and one greater spot-nosed monkey from Mambélé and two greater spot-nosed monkey samples from Somalomo in Cameroon. The remaining 16 *Oesophagostomum* haplotypes, together with other available *Oesophagostomum* spp. sequences (*n* = 45), were used to create an alignment (175 bp) to build a ML tree using model K2P + G4. Five sequences of *O. dentatum* served as the outgroup. The list of GenBank sequences used in this analysis is provided in the Additional file 2: Table S2-A.

**Fig. 3:**
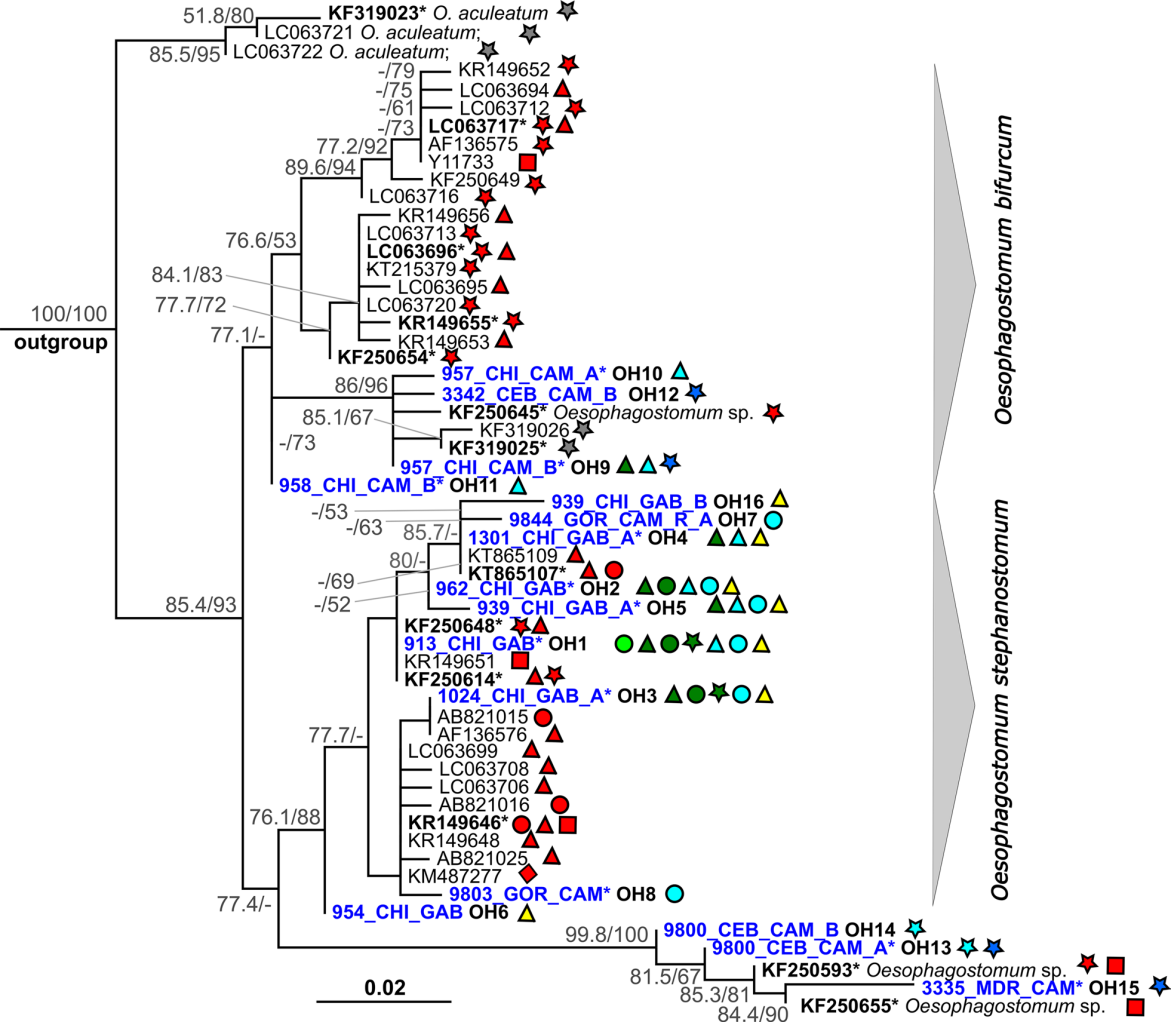
Maximum likelihood phylogenetic tree of the ITS2 region of *Oesophagostomum* spp. inferred using model K2P + G4. The phylogeny was tested by the SH-aLRT nonparametric test (left) and ultrafast bootstrap (right) in 1000 replicates. Only values > 50 (in %) are shown. Sequences originating from this study are given in blue. Sequences in bold and those marked with an asterisk indicate haplogroups comprising multiple sequences. Symbols and colour codes are as described in Fig. 2 caption

In the phylogenetic tree, haplotypes OH9–12 originating from four chimpanzees and one mangabey sample clustered in a strongly supported clade with all *O. bifurcum* sequences (Fig. 3). Our four haplotypes differed from each other by 0.6–1.1%, with the pairwise sequence distance (PSD) within the whole *O. bifurcum* clade ranging from 0.6 to 4.6%. These four haplotypes originated from Cameroon only. Haplotypes OH10 and OH11 came from three chimpanzees from Somalomo, haplotype OH12 came from a red-capped mangabey from Djoum and haplotype OH9 was detected in two chimpanzees from Somalomo and one from Mambélé, and the last one came from a red-capped mangabey from Djoum (Additional file 2: Table S2-B).

Haplotypes OH1–8 and OH16 clustered in a strongly supported clade comprising sequences of *O. stephanostomum* (Fig. 3). These haplotypes originated from all our study localities except Djoum and were detected in gorillas, chimpanzees and two agile mangabey samples (Mambélé, Cameroon) (Additional file 2: Table S2-B). These haplotypes differed from each other by 0.6–2.9%, and the overall PSD in the whole clade reached 6%. Finally, haplotypes OH13–15, which clustered with sequences of *Oesophagostomum* spp. originating from different cercopithecid monkeys and humans from Uganda, formed a clade separated and quite distant from *O. stephanostomum*, supported only by the SH-like approximate likelihood ratio test and not by bootstrap (Fig. 3). Our haplotypes originated from two red-capped mangabey samples from Somalomo and Djoum, and three mandrill samples from Djoum (Additional file 2: Table S2-B). Haplotypes OH13–15 differed by 0.6–2.9%, which is also the PSD range for the whole clade. Our haplotypes from *O. bifurcum* clade (OH9–12) differed from *O. stephanostomum* haplotypes OH1–8 and OH16 by 1.7–4.6% and from *Oesophagostomum* spp. haplotypes by 5.2–8%. A similar difference was observed between our *O. stephanostomum* and *Oesophagostomum* spp. haplotypes, varying between 4.6 and 8%.

#### *Trichuris*

**Fig. 4:**
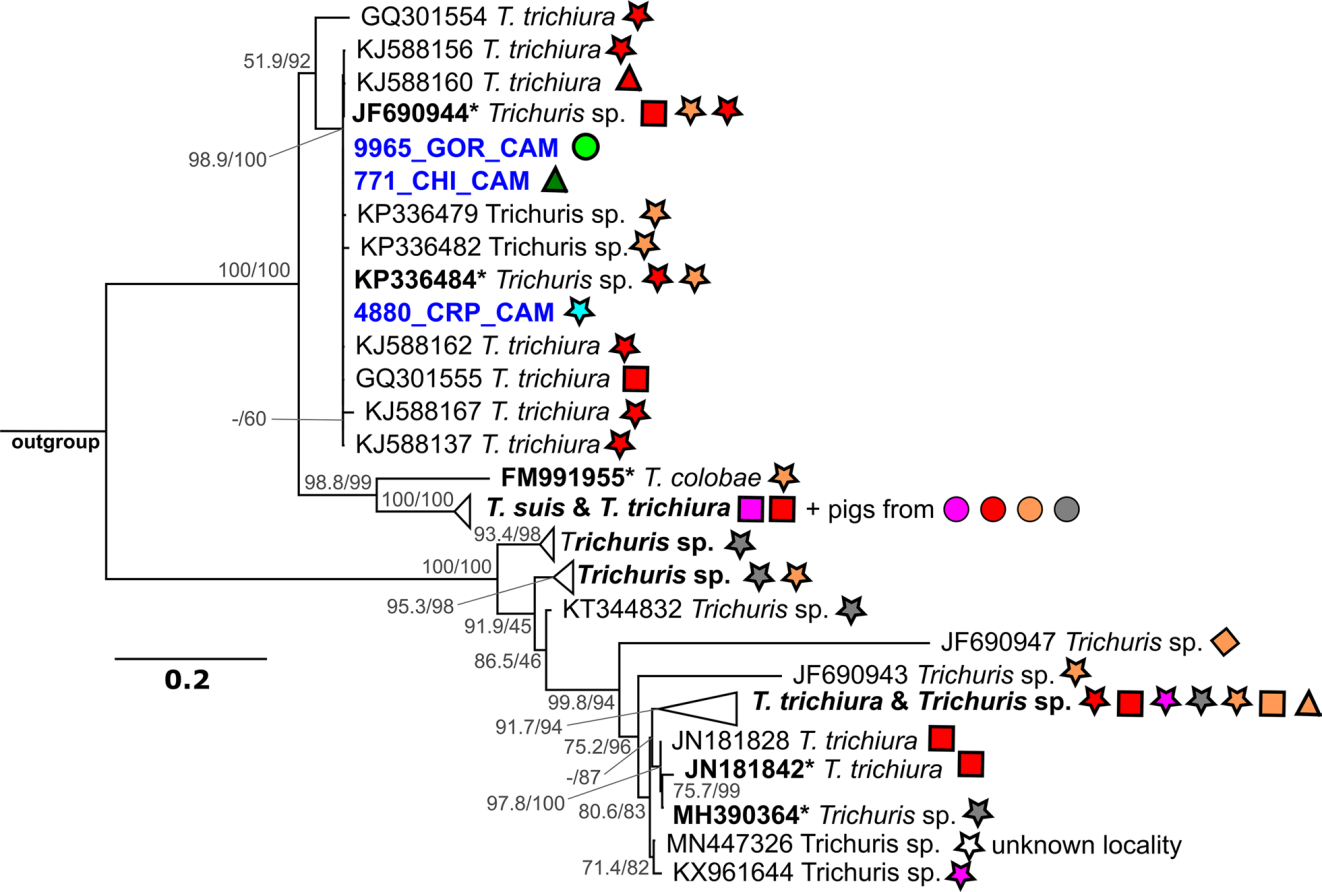
Maximum likelihood phylogenetic tree of the ITS2 region of *Trichuris* spp. inferred using the TN + F + R2 model. The phylogeny was tested by the SH-aLRT nonparametric test (left) and ultrafast bootstrap (right) in 1000 replicates. Only values > 50 (in %) are shown. Sequences originating from this study are given in blue. Sequences in bold and those marked with an asterisk indicate haplogroups comprising multiple sequences. Symbols and colour codes are as described in Fig. 2

In one PCR-positive sample, the sequencing of *Trichuris* was not successful. The region spanning the ITS1, 5.8S and ITS2 (467–703 bp) was amplified from three samples originating from a gorilla, a chimpanzee and a greater spot-nosed monkey from Lobéké, Mambélé and Somalomo, Cameroon, respectively. All three sequences were identical in the 416-bp overlapping region of ITS2. The final alignment of 625 bp from the ITS region used for phylogenetic analysis comprised 113 sequences of *Trichuris* spp. A sequence of *T. vulpis* (GenBank Accession number AM234616) was used as an outgroup. The complete list of sequences and their assignment to haplogroups is given in Additional file 2: Table S3. In the phylogenetic tree computed by the TN + F + R2 model, our sequences clustered in a strongly supported clade containing sequences of *T. trichiura* and *Trichuris* spp. originating from humans and NHP, such as *Pan troglodytes*, *Cercopithecus* spp., *Colobus* spp., *Papio* spp., *Chlorocebus aethiops* and *Procolobus rufomitratus* from Uganda, Cameroon, South Africa and some European zoos (Fig. 4; Additional file 3: Figure S1).

#### Co-infections confirmed by sequencing results

Of the 76 chimpanzees in which co-infection with *Oesophagostomum* and *Necator* was detected by our optimised PCR assay, we obtained sequences exploitable for phylogenetic analyses for 43 of them, corresponding to a 56.6%f success rate; for gorillas, we obtained only two high-quality pairs of sequences from 30 PCR-positive samples for both of these parasites. The remaining sequences were not included in the alignments used in the ML phylogenetic analyses because they were of lower quality (< 138 bp) or displayed a background noise with too many unresolved degenerated nucleotides. However, PCR results clearly displayed two separate bands corresponding to the amplicon sizes expected for *Necator* and *Oesophagostomum.*

Co-occurrence of *O. stephanostomum* and *Necator* sp. type II was quite common and was detected in 33 chimpanzees from Matakamangoye, Gabon, seven chimpanzees and one gorilla from Somalomo, Cameroon and one chimpanzee and one gorilla from Mambelé, Cameroon. Co-infection with *O. bifurcum* and *Necator* sp. type II occurred in one chimpanzee from Matakamangoye, Gabon, and in one chimpanzee from Mambelé, Cameroon. In one greater spot-nosed monkey sample from Somalomo, Cameroon, the presence of *Trichuris* sp. and *Ternidens deminutus* was detected. Finally, one gorilla in Lobéké NP, Cameroon, tested positive for *Trichuris* sp. and *Necator* sp. type II. A check-list of co-infections confirmed by sequencing is provided in Additional file 1: Table S3.

## Discussion

To the best of our knowledge, this is the first study to assess the prevalence and genetic diversity of STH in these regions of Cameroon and Gabon. Our results show that the prevalence of *Oesophagostomum* and *Necator* was relatively high in gorillas and chimpanzees; in contrast, *Trichuris* infection was low and *Ascaris* was absent. Previous studies of free-ranging gorillas from Dzanga Sangha Protected Areas, CAR and the Moukalaba-Doudou NP, Gabon, reported a similar prevalence of *Necator* and *Oesophagostomum* [27, 51]. *Trichuris* and *Ascaris* have also been detected in gorillas in Gabon and Rwanda, but at very low prevalence [51, 52]. In chimpanzees, the prevalence of *Necator* and *Oesophagostumum* was reported at relatively high rates in chimpanzee populations living in primary and secondary forest habitats in Lopé, Gabon [23]; Dzanga‐Ndoki, CAR [53]; Budongo [54], Sebitoli, [7], Kibale [6, 19, 28] and Bulindi [18, 55], Uganda; Gombe [56, 57] and Mahale [13], Tanzania. This was not the case for chimpanzees living in dry savanna habitats, where conditions for the survival and transmission of parasites with free-living stages are more difficult [53, 58]. *Trichuris* and *Ascaris* infections have been detected at a low (< 10%) or very low prevalence [23, 28, 53, 56, 59–61], with the exception of a chimpanzee community in Mahale, Tanzania [54] and one in Cantanhez NP, Republic of Guinea Bissau [62]. However, due to the variety of different detection techniques used in these studies (microscopy and molecular analyses), the results of the majority of these studies are not directly comparable, thereby making it difficult to assess and understand meaningful differences both within and between populations. In our study, *Oesophagostomum* was detected in all NHP species with the exception of the greater spot-nosed monkeys, while *Necator* was detected in the greater spot-nosed monkey and both mangabey species but not in mandrills. As we analysed only a limited number of samples from these NHP species, we were not able to compare our results with those from previously published studies and, therefore, we cannot speculate on whether arboreal monkeys have a lower exposure to infective larvae or eggs than the more terrestrial NHP. Among the NHP species we investigated, only the greater spot-nosed monkeys are almost exclusively arboreal; all of the other species are semi-terrestrial to varying degrees [63, 64], with gorillas being primarily terrestrial. Previous research reported a 41% *Oesophagostomum* prevalence in arboreal red colobus monkeys compared to 100% prevalence observed in sympatric chimpanzees, in Kibale NP, Uganda [15]. However, other factors (phylogenetic proximity, social structure and interactions, co-infection with other parasites, etc.) may contribute to determine susceptibility, as reported in the study conducted at Mahale Mountains NP, Tanzania where *Oesophagostomum* was present in all examined NHP species but prevalence was higher in arboreal red-tailed monkeys and red colobus compared to baboons [65].

We did not observe any sex difference in the prevalence of *Oesophagostomum* spp. and *Necator* spp. in chimpanzees. In this aspect, our results corroborate those of other epidemiological investigations in wild NHP [6, 27, 28, 30, 56]. However, sex differences in parasitic infection are commonly observed in many animals [32] although infection rates can fluctuate according to seasons. Other factors, such as breeding, hunting season and territory defence, can increase stress and have an impact on the immunity level of animals and on their susceptibility to parasitic infections [32, 66, 67].

In our study sites in Cameroon and in Gabon, and, in general, across an increasing number of territories, NHP are found in degraded forests and plantations or farmed landscapes near human settlements. Even national parks struggle to limit human activities, such as timber extraction, non-timber resource exploitation and hunting. In the Lobéké NP, for example, 443,825 hectares are set aside for sport hunting and another 271,945 hectares for community-managed hunting [68], and the BaAka people are allowed to go into the park to fish and harvest non-timber forest products. Somalomo, a small town (approx. 1000 inhabitants) located in the East Region of Cameroon, near the border with the Centre and South Regions, at the edge of the Dja Faunal Reserve, was the site in our study with the highest infection for both *Oesophagostomum* and *Necator* in chimpanzees and gorillas. Despite the anthropogenic pressure exerted on all territories, we did not find a single NHP infected with *Necator americanus,* a typical human parasite. However, we cannot exclude the possibility that *N. americanus* occurred in our study populations. A next-generation sequencing approach may allow the detection of co-infections with different *Necator* species and improve the quality of the sequences obtained. Possible events of zoonotic transmission of *N. americanus* have been previously reported in gorillas, chimpanzees and other NHP [69–72]. Hasegawa and colleagues [17] provided the first molecular evidence that *N. americanus* parasitises wild western lowland gorillas, but at a much lower prevalence than reported in humans; it was also the first study to report the identification of *Necator* species other than *N. americanus* in humans. These authors proposed that the worms with *cox1* of groups B or C (and ITS types II or III) are species distinct from *N. americanus* and concluded that the infective larvae examined corresponded to other hookworms previously described in the great apes, i.e., *N. congolensis*, *N. exilidens* or *N. gorillae* [73, 74]. The *Necator* we detected clearly belong to the type II species and one specimen also probably belonged to the type III species. Type II *Necator* was detected in one sample of agile mangabey in Mambélé, which expands the spectrum of hosts to cercopithecid monkeys.

For nodular worms, we found three different clades that probably represent three separate species among our surveyed NHP. The majority of our sequences belonged to nine haplotypes (OH1–OH8, plus OH16) that clustered with *O. stephanostomum.* The species was detected in both great ape species at all study sites in most of the tested samples and, in addition, in two agile mangabey samples from Mambélé. Within the study sites, we found an apparent sharing of certain haplotypes among NHP hosts, especially OH1, which was detected in all host species and at all study sites. On the other hand, it is possible that some haplotypes are site- or host-specific (e.g. OH16 was found only in Gabon; OH7 and OH8 were found only in gorillas; and OH5 was found only in chimpanzees).

Our findings confirm that *O. stephanostomum* is the predominant species in great apes [6, 7, 15, 55, 75]. This species seems to be specific to NHP, as previous studies in Gabon, Democratic Republic of the Congo and CAR failed to find zoonotic infections with *O. stephanostomum* in humans living in sympatry with infected chimpanzee or gorilla populations [10, 27, 51]. However, some zoonotic transmission may be possible, as shown by a study from Uganda [7].

A second clade clustering with *O. bifurcum* included four haplotypes (OH9–12) detected in chimpanzees and a red-capped mangabey at three study sites in Cameroon*.* Haplotype OH9 was found at all three study sites in both host species. In contrast to *O. stephanostomum*, human infection with *O. bifurcum* is well documented, but the records are mostly restricted to northern Togo and northern Ghana [76]. This parasite has a zoonotic potential, as proven in a study which demonstrated the susceptibility of rhesus monkeys to infection with *O. bifurcum* isolated from humans, albeit at low levels [77]. Interestingly, studies from Kibale NP in Uganda [7, 15] show the transmission of *O. stephanostomum* and one undetermined *Oesophagostomum* species among sympatric NHP and humans, while no *O. bifurcum* was found in humans. No *O. bifurcum* was detected in our gorilla samples, corroborating the results of a previous study from Gabon where only a single population of *O. stephanostomum*, with notable molecular variations, was prevalent in western lowland gorillas [51]. Past studies have shown that *O. bifurcum* is rarely found in great apes, but it is common in other NHP species [55]. A study conducted in the CAR reported a high prevalence of this strongylid in mangabeys but a low relative abundance in gorillas and chimpanzees [27]. Mixed infections with *O. stephanostomum* and *O. bifurcum* have been reported in chimpanzees in Bulindi NP and Kibale NP [6, 7, 55]. In our samples, we did not detect this co-infection.

Lastly, our haplotypes OH13–OH15, found in mandrills from Djoum and in a red-capped mangabey from Somalomo, Cameroon, clustered with an undetermined *Oesophagostomum* sp. These haplotypes probably represent a separate species, and they cluster with sequences sampled in a survey from Kibale, Uganda, where this *Oesophagostomum* sp. strain, shared by different cercopithecid monkeys and humans, was first reported [15]. Further studies are clearly needed to clarify the phylogenetic relationship among *Oesophagostomum* species infecting African human populations and NHP.

Four sequences, originating from a chimpanzee and one greater spot-nosed monkey from Mambélé, and two greater spot-nosed monkey samples from Somalomo, Cameroon, matched with a sequence of *Ternidens deminutus* (data not shown)*. Ternidens* infections of monkeys, chimpanzees, gorillas and baboons have been reported throughout sub-Saharan Africa and Asia [27, 78]. *Ternidens deminutus* is thought to be a zoonosis acquired from NHP, although some “spillback” from humans to monkey populations may also occur in some areas [79]. The possible presence of multiple cryptic species has been suggested, with a human-specific haplotype of the parasite existing alongside several host-specific NHP genotypes. This theory is supported by the analogous presence of several host-specific haplotypes, including a human-specific haplotype, in the genetically similar helminth species *O. bifurcum* [80]. Ternidensiasis most commonly has a similar clinical picture as oesophagostomiasis, with multiple intestinal abscess nodules or helminthomas of the large intestine [79].

Finally, we found evidence of a significant overabundance of co-infections with *Oesophagostomum* and *Necator*, corroborating previous observations of co-infections in chimpanzees with hookworms and nodular worms in Uganda [18, 27]. Indeed, the relative overabundance of parasite co-infection appears to be common in many host species [81, 82]. In fact, our results may underestimate the actual number of co-infections because conventional PCR followed by Sanger sequencing has its limitations in terms of discriminating between the presence of multiple strongylid species/lineages in a single sample. The low rate of high-quality sequences we obtained may also be explained by the presence of parasite co-infections in the samples analysed.

Regarding the health impact of these STH on their host, hookworms produce wounds that will constantly bleed by using their buccal capsules to attach to the mucosal surfaces of the host’s small intestine [83, 84]. In addition to blood loss, this process is responsible for secondary bacterial infections and significant inflammation in the mucosae, impairing digestion and absorption [85]. Therefore, the main adverse effects of these hematophagous parasites recorded in humans, domestic animals and wildlife species are anaemia, retarded growth, secondary bacteraemia and mortality [83, 85–88]. *Oesophagostomum* infections are responsible for oesophagostomiasis, an uninodular or multinodular disease with symptoms in NHP raised in sanctuaries (acute weakness and abdominal pain, vomiting, diarrhoea and weight loss) that are similar to those recorded in severely ill human patients [89]. Bowel obstruction, nodule rupture resulting in peritonitis, hepatic complications, among others are the signs observed during post-mortem examinations of captive chimpanzees and gorillas [90, 91]. In the wild, the health impact of nodular worm infection in NHP has been found to be variable. On the one hand, pathologic lesions associated with *Oesophagostomum* spp. infection have been described in wild chimpanzees from Kibale NP and Mahale in Uganda (*Pan troglodytes schweinfurthii*), Taï NP in Cote d’Ivoire (*Pan troglodytes verus*) and Gombe NP in Tanzania (*Pan troglodytes schweinfurthii*) [13, 14, 92]. In Gombe NP, chimpanzees suffered from weight loss and other clinical signs suggestive of significant infections [14]. On the other hand, in other NHP populations, *Oesophagostomum* infections did not appear to affect the host’s overall health [92]. With the exceptions mentioned above, the rarity of overt clinical signs of disease linked to specific intestinal parasites in wild apes suggests that infections by many species are tolerated and/or mediated by self-medicative behaviours, such as leaf swallowing [13, 18, 92, 93]. Clinical differences between free-ranging and (semi-) captive populations could be explained by differences in stress level, limited space, proximity of individuals, deworming procedures, infection with human strains, closer monitoring and the easier diagnosis in NHP living in sanctuaries [92]. Given the perspective of a shrinking natural habitat combined with the increasing sharing of habitat between humans and NHP, these pathologies represent a major threat to human and NHP health.

Among the 315 samples tested, only four (one gorilla, two chimpanzees and a greater spot-nosed monkey) were positive for *Trichuris* sp. Three sequences were exploitable and clustered together with *T. trichiura* and *Trichuris* spp. originating from humans and other NHP. The low prevalence detected is comparable with that recorded for chimpanzees in many other localities [13, 23, 60, 94]. However, some notable exceptions of high prevalence of infection have been reported in chimpanzee populations in Cantanhez NP in Guinea-Bissau (15% prevalence) and at Mont Assirik in Senegal (50% prevalence) [62, 95]. Other studies focused primarily on the genetic diversity of this nematode, mostly from captive settings with only a few more reporting low prevalence in the wild NHP populations studied [16, 21, 96, 97]. For a long time, *T. trichiura* has been described as a species with low host specificity, infecting human and NHP alike; recent studies have brought to light the existence of multiple lineages, probably cryptic species, of *Trichuris* that seem to be more host specific than expected [98]. Additionally, two species of *Trichuris* were recently described in NHP, namely *T. colobae* from *Colobus guereza kikuyensis* [99] and *T. ursinus* from *Papio ursinus* [100].

We did not detect any *Ascaris* sp. in any of the wild NHP species analysed, corroborating the results of previous research. Roundworms of the genus *Ascaris* are frequently found in captive chimpanzees but are not common in their wild counterparts [101, 102]. Altogether, parasites such as *Trichuris* and *Ascaris* seem to be almost absent (or occur at low prevalence) in the wild, contrary to what has been observed in captive gorillas both in zoos and sanctuaries [103].

## Conclusions

This study reports new information on the occurrence and genetic diversity of STH infecting six different NHP species living in Cameroon and Gabon. Spillover and spillback processes may play an important role in the maintenance of STH burden at the human–wildlife interface. Our results highlight the need for a better understanding of potential parasite-sharing between humans and NHP. On the one hand, a sylvatic STH reservoir could increases the risk of infection, particularly among human populations living in close proximity to NHP; on the other hand, proximity to human settlements may pose a threat to NHP health by facilitating the transmission of STH species or strains with lower host specificity. For STH diagnostics, molecular assays appear to be more sensitive for species identification than microscopy, particularly at low infection intensities [104, 105]. To demonstrate similarities and differences in STH infecting humans and NHP, extensive sequencing of both human and NHP isolates will need to be performed. While current molecular assays targeting ribosomal and mitochondrial sequences have vastly improved the sensitivity of the diagnosis of STH infections, some DNA markers often lack the specificity required to discriminate between different species within the same genus, which is crucial in studies of parasite-sharing across different host species. The recent development of next-generation sequencing-based DNA analysis applicable to primate parasites [106–108] allows these complex communities to be identified and described; moreover, this methodology approach should be used more extensively, as it is capable of detecting rare and cryptic species*.* In conclusion, calls for better control of STH require an improved understanding of their epidemiology, genetic diversity and degree of zoonotic transmission.

## Supplementary Information

**Additional file 1: Table S1.** Number of chimpanzees, gorillas or the two species combined, positive or negative for* Oesophagostomum* spp. co-infected or not with* Necator* spp. (values under the null hypothesis of co-infections occurring more often than expected by chance are shown in parentheses). **Table S2.** Number of male and female chimpanzees positive or negative for* Oesophagostomum* spp. and for* Necator* spp. (expected values under the null hypothesis of no sex difference are shown in parentheses). **Table S3.** Check-list of co-infections confirmed by sequencing.

**Additional file 2: Table S1-A.** List of *Necator* spp. ITS2 sequences from GenBank used in this study and their assignment to haplogroups, together with the information on the host and locality. The sequences in bold are representatives for the given haplogroup and were used in the phylogenetic tree. **Table S1-B.** List of *Necator* spp. ITS2 sequences generated in this study and their assignment to the haplogroups, together with the information on the host and locality. **Table S2-A.** List of *Oesophagostomum* spp. ITS2 sequences from GenBank used in this study and their assignment to haplogroups, together with the information on the host and locality. The sequences in bold are representatives for the given haplogroup and were used in the phylogenetic tree. **Table S2-B**. List of *Oesophagostomum* spp. ITS2 sequences generated in this study and their assignment to the haplogroups, together with the information on the host and locality. The sequences in bold are representatives for the given haplogroup and were used in the phylogenetic tree. **Table S3.** List of *Trichuris* spp. ITS2 sequences from GenBank, the sequences from this study and their assignment to the haplogroups, together with the information on the host and locality. The sequences in bold are representatives for the given haplogroup and were used in the phylogenetic tree.

**Additional file 3: Figure S1.** Maximum likelihood phylogenetic tree of ITS2 region of *Trichuris*-non-collapsed.

## Data Availability

All data generated or analysed during this study are included in this published article (and its Additional files). We deposited all newly generated sequences from this study in GenBank under accession numbers MW528535–MW528543 and MW621486–MW621493 for *Necator* spp., MW528465–MW528480 and MW621343–MW621355 for *Oesophagostomum* spp., MW528545–MW528547 for *Trichuris* sp. and MW617064–MW617066 for *Ternidens deminutus.*

## Abbreviations

BIC: Bayesian information criterion
BLASTn: Nucleotide Basic Local Alignment Search Tool
CAR: Central African Republic
DJ: Djoum
ITS (1, 2): Internal transcribed spacer (1, 2)
LB: Lobéké National Park
MB: Mambélé
MKT: Matakamangoye
mtDNA: Mitochondrial DNA
NHP: Non-human primates
NP: National Park
PSD: Pairwise sequence distance
SIV: Simian immunodeficiency virus
SOM: Somalomo
STH: Soil-transmitted helminths
