# Supplementary material for: Soil-transmitted helminth infections in free-ranging non-human primates from Cameroon and Gabon
Source: Parasit Vectors. 2021 Jul 5;14:354. doi: 10.1186/s13071-021-04855-7 (PMC8259424; doi:10.1186/s13071-021-04855-7)
Supplement: Supplementary file 1 — Additional file 1: Table S1. Number of chimpanzees, gorillas or the two species combined, positive or negative for Oesophagostomum spp. co-infected or not with Necator spp. (values under the null hypothesis of co-infections occurring more often than expected by chance are shown in parentheses). Table S2. Number of male and female chimpanzees positive or negative for Oesophagostomum spp. and for Necator spp. (expected values under the null hypothesis of no sex difference are shown in parentheses). Table S3. Check-list of co-infections confirmed by sequencing. [file 13071_2021_4855_MOESM1_ESM.docx]

**Table S1** Number of chimpanzees, gorillas or the two species combined, positive or negative for *Oesophagostomum* sp. coinfected or not with *Necator* sp. (values under the null hypothesis of co-infections occurring more often than expected by chance, are shown in parentheses)

| Chimpanzees |  | *Necator* sp. | | |  |
| --- | --- | --- | --- | --- | --- |
|  |  | Positive | Negative | Total | Two-tailed probability |
| *Oesophagostomum* sp. | Positive | 76 (70.7) | 81 (86.3) | 157 | 0.139 |
|  | Negative | 24 (29.3) | 41 (35.7) | 65 |  |
|  | Total | 100 | 122 | 222 |  |
|  |  |  |  |  |  |
| Gorillas |  | *Necator* sp. | | |  |
|  |  | Positive | Negative | Total | Two-tailed probability |
| *Oesophagostomum* sp. | Positive | 30 (27.5) | 21 (23.5) | 51 | 0.143 |
|  | Negative | 5 (7.5) | 9 (6.5) | 14 |  |
|  | Total | 35 | 30 | 65 |  |
|  |  |  |  |  |  |
| Chimpanzees and Gorillas |  | *Necator* sp. | | |  |
|  |  | Positive | Negative | Total | Two-tailed probability |
| *Oesophagostomum* sp*.* | Positive | 107 (98.3) | 102 (110.7) | 209 | 0.023 |
|  | Negative | 28 (36.7) | 50 (41.3) | 78 |  |
|  | Total | 135 | 152 | 287 |  |

**Table S2** Number of female and male chimpanzees positive or negative for *Oesophagostomum* sp. and for *Necator* sp. and comparison of prevalence between sexes (expected frequencies per null hypothesis in parenthesis)

| Parasite species | Sex | No. positives (%) | No. negatives | Total | P-value |
| --- | --- | --- | --- | --- | --- |
| *Oesophagostomum* sp. | Females | 32 (80.0) | 8 | 40 | 0.645 |
|  | Males | 55 (74.3) | 19 | 74 |  |
|  | Total | 87 (73.3) | 27 | 114 |  |
| *Necator* sp. | Females | 16 (40.0) | 24 | 40 | 0.326 |
|  | Males | 38 (51.4) | 36 | 74 |  |
|  | Total | 54 (47.4) | 60 | 114 |  |

**Table S3** Check-list of co-infections confirmed by sequencing

| **Host and location** | ***Necator* Type II** | ***O. stephanostomum*** | ***O. bifurcum*** | ***Trichuris* sp.** | ***Ternidens deminutus*** |
| --- | --- | --- | --- | --- | --- |
| **Chimpanzee (N=33)** Matakamangoye (GAB) | x | x |  |  |  |
| **Chimpanzee (N=1)** Somalomo (CAM) | x |  | x |  |  |
| **Chimpanzee (N=7)** Somalomo (CAM) | x | x |  |  |  |
| **Gorilla** (N=1) Somalomo (CAM) | x | x |  |  |  |
| **Greater spot-nosed monkey (N=1)** Somalomo (CAM) |  |  |  | x | x |
| **Agile mangabey** **(N=1)** Mambélé (CAM) | x | x |  |  |  |
| **Chimpanzee (N=1)** Mambélé (CAM) | x |  |  | x |  |
| **Chimpanzee (N=1)**  Mambélé (CAM) | x |  |  |  |  |
| **Chimpanzee (N=1)** Mambélé (CAM) | x |  | x |  |  |
| **Gorilla (N=1)** Mambélé (CAM) | x | x |  |  |  |
| **Gorilla (N=1)** Lobéké NP | x |  |  | x |  |
