# Supplementary material for: Soil-transmitted helminth infections in free-ranging non-human primates from Cameroon and Gabon
Source: Parasit Vectors. 2021 Jul 5;14:354. doi: 10.1186/s13071-021-04855-7 (PMC8259424; doi:10.1186/s13071-021-04855-7)
Supplement: Supplementary file 2 — Additional file 2: Table S1-A. List of Necator spp. ITS2 sequences from GenBank used in this study and their assignment to haplogroups, together with the information on the host and locality. The sequences in bold are representatives for the given haplogroup and were used in the phylogenetic tree. Table S1-B. List of Necator spp. ITS2 sequences generated in this study and their assignment to the haplogroups, together with the information on the host and locality. Table S2-A. List of Oesophagostomum spp. ITS2 sequences from GenBank used in this study and their assignment to haplogroups, together with the information on the host and locality. The sequences in bold are representatives for the given haplogroup and were used in the phylogenetic tree. Table S2-B. List of Oesophagostomum spp. ITS2 sequences generated in this study and their assignment to the haplogroups, together with the information on the host and locality. The sequences in bold are representatives for the given haplogroup and were used in the phylogenetic tree. Table S3. List of Trichuris spp. ITS2 sequences from GenBank, the sequences from this study and their assignment to the haplogroups, together with the information on the host and locality. The sequences in bold are representatives for the given haplogroup and were used in the phylogenetic tree. [file 13071_2021_4855_MOESM2_ESM.docx]

**Table S1-A**

List of *Necator* ITS2 sequences from GenBank used in this study and their assignment to haplogroups, together with the information on the host and locality. The sequences in bold are representatives for the given haplogroup and were used in the phylogenetic tree.

| **Haplogroup** | **Sequence** | **Organism** | **Host** | **Locality** |
| --- | --- | --- | --- | --- |
| 1 | **AB793530** | *Necator* sp. | *Homo sapiens* | CAR |
|  | LC088241 | *Necator* sp. | *P. troglodytes* | Uganda |
|  | LC088242 | *Necator* sp. | *P. troglodytes* | Uganda |
|  | LC088245 | *Necator* sp. | *P. troglodytes* | Uganda |
|  | LC088247 | *Necator* sp. | *P. troglodytes* | Uganda |
|  | LC088252 | *Necator* sp. | *P. troglodytes* | Uganda |
|  | LC088254 | *Necator* sp. | *P. troglodytes* | Uganda |
|  | LC088256 | *Necator* sp. | *P. troglodytes* | Uganda |
|  | LC088257 | *Necator* sp. | *P. troglodytes* | Uganda |
|  | LC088258 | *Necator* sp. | *P. troglodytes* | Uganda |
|  | LC088261 | *Necator* sp. | *P. troglodytes* | Uganda |
|  | LC088263 | *Necator* sp. | *P. troglodytes* | Uganda |
|  | LC088265 | *Necator* sp. | *P. troglodytes* | Uganda |
|  | LC088266 | *Necator* sp. | *P. troglodytes* | Uganda |
|  | LC088267 | *Necator* sp. | *P. troglodytes* | Uganda |
|  | LC088268 | *Necator* sp. | *P. troglodytes* | Uganda |
|  | LC088275 | *Necator* sp. | *P. troglodytes* | Uganda |
|  | LC088277 | *Necator* sp. | *P. troglodytes* | Uganda |
|  | LC088280 | *Necator* sp. | *P. troglodytes* | Uganda |
|  | LC088281 | *Necator* sp. | *P. troglodytes* | Uganda |
|  | LC088282 | *Necator* sp. | *P. troglodytes* | Uganda |
|  | LC088283 | *Necator* sp. | *P. troglodytes* | Uganda |
|  | LC088285 | *Necator* sp. | *P. troglodytes* | Uganda |
|  | AB793529 | *Necator* sp. | *Homo sapiens* | CAR |
|  | LC088259 | *Necator* sp. | *P. troglodytes* | Uganda |
|  | LC088298 | *Necator* sp. | *G. g. gorilla* | Gabon |
|  | LC088299 | *Necator* sp. | *G. g. gorilla* | Gabon |
|  | LC088269 | *Necator* sp. | *P. troglodytes* | Uganda |
|  | LC088243 | *Necator* sp. | *P. troglodytes* | Uganda |
|  | LC088250 | *Necator* sp. | *P. troglodytes* | Uganda |
|  | AB793533 | *Necator* sp. | *Homo sapiens* | CAR |
| 2 | **LC088240** | *Necator* sp. | *P. troglodytes* | Uganda |
| 3 | **LC088248** | *Necator* sp. | *P. troglodytes* | Uganda |
| 4 | **LC088278** | *Necator* sp. | *P. troglodytes* | Uganda |
| 5 | **LC088262** | *Necator* sp. | *P. troglodytes* | Uganda |
| 6 | **AB793534** | *Necator* sp. | *G. g. gorilla* | CAR |
| 7 | **AB793535** | *Necator* sp. | *Homo sapiens* | CAR |
| 8 | **KM487279** | *Necator* sp. | *P. paniscus* | DRC |
| 9 | **MG770106** | *N. americanus* | *Homo sapiens* | Ghana |
|  | MG770099 | *N. americanus* | *Homo sapiens* | Ghana |
|  | MG770101 | *N. americanus* | *Homo sapiens* | Ghana |
|  | MG770105 | *N. americanus* | *Homo sapiens* | Ghana |
|  | MG770107 | *N. americanus* | *Homo sapiens* | Ghana |
|  | MH665842 | *N. americanus* | *Homo sapiens* | Brazil |
|  | AB793527 | *N. americanus* | *Homo sapiens* | CAR |
|  | AF217891 | *N. americanus* | *Homo sapiens* | Guatemala |
|  | KM487278 | *N. americanus* | *Homo sapiens* | DRC |
| 10 | **KC632571** | *N. americanus* | *Homo sapiens* | Australia |
|  | MG770100 | *N. americanus* | *Homo sapiens* | Ghana |
|  | MG770104 | *N. americanus* | *Homo sapiens* | Ghana |
| 11 | **HQ452543** | *N. americanus* | *Homo sapiens* | Malaysia |
|  | LC036563 | *N. americanus* | *Homo sapiens* | Japan |
|  | JF960370 | *N. americanus* | *Homo sapiens* | Malaysia |
|  | JF960374 | *N. americanus* | *Homo sapiens* | Malaysia |
|  | JF960387 | *N. americanus* | *Homo sapiens* | Malaysia |
|  | JF960388 | *N. americanus* | *Homo sapiens* | Malaysia |
|  | JF960392 | *N. americanus* | *Homo sapiens* | Malaysia |
|  | JF960395 | *N. americanus* | *Homo sapiens* | Malaysia |
|  | JF960401 | *N. americanus* | *Homo sapiens* | Malaysia |
| 12 | **JF960376** | *N. americanus* | *Homo sapiens* | Malaysia |
|  | JF960375 | *N. americanus* | *Homo sapiens* | Malaysia |
| 13 | **JF960400** | *N. americanus* | *Homo sapiens* | Malaysia |
|  | HQ452537 | *N. americanus* | *Homo sapiens* | Malaysia |
|  | HQ452539 | *N. americanus* | *Homo sapiens* | Malaysia |
|  | HQ452540 | *N. americanus* | *Homo sapiens* | Malaysia |
|  | HQ452541 | *N. americanus* | *Homo sapiens* | Malaysia |
|  | HQ452542 | *N. americanus* | *Homo sapiens* | Malaysia |
|  | KM891738 | *N. americanus* | N/A | N/A |
|  | KC632569 | *N. americanus* | *Homo sapiens* | Australia |
|  | KU513968 | *N. americanus* | *Homo sapiens* | Iran |
|  | JF960373 | *N. americanus* | *Homo sapiens* | Malaysia |
|  | JF960377 | *N. americanus* | *Homo sapiens* | Malaysia |
|  | JF960382 | *N. americanus* | *Homo sapiens* | Malaysia |
|  | JF960384 | *N. americanus* | *Homo sapiens* | Malaysia |
|  | JF960385 | *N. americanus* | *Homo sapiens* | Malaysia |
|  | JF960389 | *N. americanus* | *Homo sapiens* | Malaysia |
|  | JF960391 | *N. americanus* | *Homo sapiens* | Malaysia |
|  | JF960396 | *N. americanus* | *Homo sapiens* | Malaysia |
|  | JF960397 | *N. americanus* | *Homo sapiens* | Malaysia |
|  | JF960399 | *N. americanus* | *Homo sapiens* | Malaysia |
| 14 | **JF960380** | *N. americanus* | *Homo sapiens* | Malaysia |
| 15 | **JF960381** | *N. americanus* | *Homo sapiens* | Malaysia |
| 16 | **LC088287** | *N. americanus* | *G. g. gorilla* | Gabon |
| 17 | **Y11734** | *N. americanus* | *Homo sapiens* | Togo |
| 18 | **MG770102** | *N. americanus* | *Homo sapiens* | Ghana |
| 19 | **MG770103** | *N. americanus* | *Homo sapiens* | Ghana |
| 20 | **MK036419** | *N. americanus* | *Homo sapiens* | China |
| 21 | **JF960383** | *N. americanus* | *Homo sapiens* | Malaysia |
| 22 | **JF960372** | *N. americanus* | *Homo sapiens* | Malaysia |
| 23 | **AJ001600** | *N. americanus* | *Homo sapiens* | Malaysia |
| 24 | **JF960393** | *N. americanus* | *Homo sapiens* | Malaysia |
| 25 | **JF960390** | *N. americanus* | *Homo sapiens* | Malaysia |
| 26 | **JF960378** | *N. americanus* | *Homo sapiens* | Malaysia |
| 27 | **LC036565** | *N. americanus* | *Homo sapiens* | Laos |
| 28 | **KU855047** | *N. americanus* | soil | Thailand |
| 29 | **JF960386** | *N. americanus* | *Homo sapiens* | Malaysia |
| 30 | **JF960403** | *N. americanus* | *Homo sapiens* | Malaysia |
| 31 | **KU855048** | *N. americanus* | soil | Thailand |

N/A – not available

CAR – Central African Republic

DRC – Democratic Republic of the Congo

**Table S1-B**

List of *Necator* ITS2 sequences generated in this study and their assignment to the haplogroups, together with the information on the host and locality. The sequences in bold are representatives for the given haplogroup and were used in the phylogenetic tree.

| **Variant** | **Sequence** | **Accession number** | **Host** | **Locality** |
| --- | --- | --- | --- | --- |
| **NH1** (38 T, 228 T, 265A) | **2352_CHI_GAB** | MW528542 | *P. t. troglodytes* | Gabon, MKT |
| (n= 14) | 951_CHI_GAB |  | *P. t. troglodytes* | Gabon, MKT |
|  | 962_CHI_GAB |  | *P. t. troglodytes* | Gabon, MKT |
|  | 1279_CHI_GAB |  | *P. t. troglodytes* | Gabon, MKT |
|  | 1298_CHI_GAB |  | *P. t. troglodytes* | Gabon, MKT |
|  | 1299_CHI_GAB |  | *P. t. troglodytes* | Gabon, MKT |
|  | 1332L_CHI_GAB |  | *P. t. troglodytes* | Gabon, MKT |
|  | 1342_CHI_GAB |  | *P. t. troglodytes* | Gabon, MKT |
|  | 1347_CHI_GAB |  | *P. t. troglodytes* | Gabon, MKT |
|  | 2334_CHI_GAB |  | *P. t. troglodytes* | Gabon, MKT |
|  | 2347_CHI_GAB |  | *P. t. troglodytes* | Gabon, MKT |
|  | 2496_GOR_CAM |  | *G. g. gorilla* | Cameroon, LB |
|  | 5151_CHI_CAM | MW621489 | *P. t. troglodytes* | Cameroon, MB |
|  | 9964_GOR_CAM | MW621492 | *G. g. gorilla* | Cameroon, LB |
| **NH2** (38 T, 22 T, 265 T) | **941_CHI_GAB** | MW528538 | *P. t. troglodytes* | Gabon, MKT |
| (n= 32) | **770_CHI_CAM** | MW528539 | *P. t. troglodytes* | Cameroon, MB |
|  | 771_CHI_CAM |  | *P. t. troglodytes* | Cameroon, MB |
|  | 776_CHI_CAM |  | *P. t. troglodytes* | Cameroon, MB |
|  | 888_CHI_CAM |  | *P. t. troglodytes* | Cameroon, MB |
|  | 932_CHI_GAB |  | *P. t. troglodytes* | Gabon, MKT |
|  | 934_CHI_GAB |  | *P. t. troglodytes* | Gabon, MKT |
|  | 939_CHI_GAB |  | *P. t. troglodytes* | Gabon, MKT |
|  | 956_CHI_CAM |  | *P. t. troglodytes* | Cameroon, SOM |
|  | 957_CHI_CAM | MW621486 | *P. t. troglodytes* | Cameroon, SOM |
|  | 960_CHI_CAM |  | *P. t. troglodytes* | Cameroon, SOM |
|  | 987_CHI_GAB |  | *P. t. troglodytes* | Gabon, MKT |
|  | 990_CHI_GAB |  | *P. t. troglodytes* | Gabon, MKT |
|  | 998_CHI_GAB |  | *P. t. troglodytes* | Gabon, MKT |
|  | 1002_CHI_GAB |  | *P. t. troglodytes* | Gabon, MKT |
|  | 1008_CHI_GAB |  | *P. t. troglodytes* | Gabon, MKT |
|  | 1015_CHI_GAB |  | *P. t. troglodytes* | Gabon, MKT |
|  | 1021_CHI_GAB |  | *P. t. troglodytes* | Gabon, MKT |
|  | 1022_CHI_GAB |  | *P. t. troglodytes* | Gabon, MKT |
|  | 1169_CHI_GAB |  | *P. t. troglodytes* | Gabon, MKT |
|  | 1179_CHI_GAB |  | *P. t. troglodytes* | Gabon, MKT |
|  | 1237_CHI_GAB |  | *P. t. troglodytes* | Gabon, MKT |
|  | 1250_CHI_GAB |  | *P. t. troglodytes* | Gabon, MKT |
|  | 1296_CHI_GAB |  | *P. t. troglodytes* | Gabon, MKT |
|  | 1334_CHI_GAB |  | *P. t. troglodytes* | Gabon, MKT |
|  | 1343_CHI_GAB |  | *P. t. troglodytes* | Gabon, MKT |
|  | 1464_CHI_GAB |  | *P. t. troglodytes* | Gabon, MKT |
|  | 2340_CHI_GAB |  | *P. t. troglodytes* | Gabon, MKT |
|  | 2349_CHI_GAB |  | *P. t. troglodytes* | Gabon, MKT |
|  | 2364_CHI_GAB |  | *P. t. troglodytes* | Gabon, MKT |
|  | 2366_CHI_GAB |  | *P. t. troglodytes* | Gabon, MKT |
|  | 8386_CHI_CAM |  | *P. t. troglodytes* | Cameroon, MB |
| **NH3** (38 C, 228 Y, 265 N/A) | **6954_CHI_CAM** | MW528536 | *P. t. troglodytes* | Cameroon, MB |
| **NH4** (38 Y, 228 A, 265 T) | **5135_CHI_CAM** | MW528540 | *P. t. troglodytes* | Cameroon, MB |
| **NH5** | **945_CHI_GAB** | MW528535 | *P. t. troglodytes* | Gabon, MKT |
| **NH1** or **NH2** | **1297_CHI_GAB** | MW528541 | *P. t. troglodytes* | Gabon, MKT |
| (n= 23) | **9965_GOR_CAM** | MW528537 | *G. g. gorilla* | Cameroon, LB |
|  | 889_CHI_CAM |  | *P. t. troglodytes* | Cameroon, MB |
|  | 937_CHI_GAB |  | *P. t. troglodytes* | Gabon, MKT |
|  | 962_CHI_CAM | MW621487 | *P. t. troglodytes* | Cameroon, SOM |
|  | 963_CHI_CAM |  | *P. t. troglodytes* | Cameroon, SOM |
|  | 965_CHI_CAM |  | *P. t. troglodytes* | Cameroon, SOM |
|  | 984_CHI_CAM |  | *P. t. troglodytes* | Cameroon, SOM |
|  | 988_CHI_GAB |  | *P. t. troglodytes* | Gabon, MKT |
|  | 1214_CHI_GAB |  | *P. t. troglodytes* | Gabon, MKT |
|  | 1241_CHI_GAB |  | *P. t. troglodytes* | Gabon, MKT |
|  | 1243_CHI_GAB |  | *P. t. troglodytes* | Gabon, MKT |
|  | 1253_CHI_GAB |  | *P. t. troglodytes* | Gabon, MKT |
|  | 1267_CHI_GAB |  | *P. t. troglodytes* | Gabon, MKT |
|  | 1275_CHI_GAB |  | *P. t. troglodytes* | Gabon, MKT |
|  | 1291_CHI_GAB |  | *P. t. troglodytes* | Gabon, MKT |
|  | 1320_CHI_GAB |  | *P. t. troglodytes* | Gabon, MKT |
|  | 1336_CHI_GAB |  | *P. t. troglodytes* | Gabon, MKT |
|  | 1344_CHI_GAB |  | *P. t. troglodytes* | Gabon, MKT |
|  | 2318_CHI_GAB |  | *P. t. troglodytes* | Gabon, MKT |
|  | 5150_AGILIS_CAM | MW621488 | *C. agilis* | Cameroon, MB |
|  | 6949_CHI_CAM | MW621490 | *P. t. troglodytes* | Cameroon, MB |
|  | 10017_GOR_CAM | MW621493 | *G. g. gorilla* | Cameroon, MB |
| **NH1, NH2** or **NH4** | **966_CHI_CAM** | MW528543 | *P. t. troglodytes* | Cameroon, SOM |
|  | 4877_CHI_CAM |  | *P. t. troglodytes* | Cameroon, SOM |
|  | 9824_GOR_CAM | MW621491 | *G. g. gorilla* | Cameroon, SOM |

MB – Mambélé; MKT – Makamangoye; LB – Lobéké National Park; SOM – Somalomo. Positions are numbered based on the alignment of all obtained sequences.

**Table S2-A**

List of *Oesophagostomum* spp. ITS2 sequences from GenBank used in this study and their assignment to haplogroups (HG), together with the information on host and locality. The sequences in bold are representatives for the given haplogroup and were used in the phylogenetic tree.

| **HG** | **Sequence** | **Organism** | **Host** | **Locality** |
| --- | --- | --- | --- | --- |
| 1. | **KM487277.1** | *O. stephanostomum* | *Pan paniscus* | DRC |
| 2. | **KF250614.1** | *O. stephanostomum* | *Cercopithecus mitis* | Uganda |
|  | KF250588.1 | *O. stephanostomum* | *Cercopithecus ascanius* | Uganda |
|  | KF250594.1 | *O. stephanostomum* | *Cercopithecus ascanius* | Uganda |
|  | KF250598.1 | *O. stephanostomum* | *Colobus guereza* | Uganda |
|  | KF250599.1 | *O. stephanostomum* | *Colobus guereza* | Uganda |
|  | KF250612.1 | *O. stephanostomum* | *Cercopithecus ascanius* | Uganda |
|  | KF250613.1 | *O. stephanostomum* | *Cercopithecus ascanius* | Uganda |
|  | KF250615.1 | *O. stephanostomum* | *Cercopithecus mitis* | Uganda |
|  | KF250616.1 | *O. stephanostomum* | Red colobus | Uganda |
|  | KF250617.1 | *O. stephanostomum* | Red colobus | Uganda |
|  | KF250618.1 | *O. stephanostomum* | *Cercopithecus ascanius* | Uganda |
|  | KF250619.1 | *O. stephanostomum* | *Cercopithecus ascanius* | Uganda |
|  | KF250631.1 | *O. stephanostomum* | *Pan troglodytes* | Uganda |
|  | KF250634.1 | *O. stephanostomum* | *Cercopithecus ascanius* | Uganda |
|  | KF250635.1 | *O. stephanostomum* | *Cercopithecus ascanius* | Uganda |
|  | KF250637.1 | *O. stephanostomum* | *Cercopithecus ascanius* | Uganda |
|  | KF250638.1 | *O. stephanostomum* | Red colobus | Uganda |
|  | KF250643.1 | *O. stephanostomum* | *Pan troglodytes* | Uganda |
|  | KF250653.1 | *O. stephanostomum* | *Cercopithecus mitis* | Uganda |
|  | KR149649.1 | *O. stephanostomum* | *P. t. schweinfurthii* | Uganda |
|  | KR149650.1 | *O. stephanostomum* | *Colobus guereza* | Uganda |
|  | KT865106.1 | *O. stephanostomum* | *Pan troglodytes* | Tanzania |
|  | KT865108.1 | *O. stephanostomum* | *Pan troglodytes* | Tanzania |
| 3. | **KF250648.1** | *O. stephanostomum* | *Cercopithecus ascanius* | Uganda |
|  | KF250585.1 | *O. stephanostomum* | *Cercopithecus ascanius* | Uganda |
|  | KF250586.1 | *O. stephanostomum* | *Cercopithecus ascanius* | Uganda |
|  | KF250592.1 | *O. stephanostomum* | *Cercopithecus ascanius* | Uganda |
|  | KF250595.1 | *O. stephanostomum* | *Lophocebus albigena* | Uganda |
|  | KF250605.1 | *O. stephanostomum* | *Cercopithecus mitis* | Uganda |
|  | KF250606.1 | *O. stephanostomum* | *Cercopithecus mitis* | Uganda |
|  | KF250639.1 | *O. stephanostomum* | *Pan troglodytes* | Uganda |
|  | KF250640.1 | *O. stephanostomum* | *Pan troglodytes* | Uganda |
|  | KF250641.1 | *O. stephanostomum* | *Pan troglodytes* | Uganda |
|  | KF250642.1 | *O. stephanostomum* | *Pan troglodytes* | Uganda |
|  | KF250644.1 | *O. stephanostomum* | *Lophocebus albigena* | Uganda |
|  | KF250647.1 | *O. stephanostomum* | *Pan troglodytes* | Uganda |
| 4. | **KR149651.1** | *O. stephanostomum* | *Homo sapiens* | Uganda |
| 5. | **KT865109.1** | *O. stephanostomum* | *Pan troglodytes* | Tanzania |
| 6. | **KT865107.1** | *O. stephanostomum* | *Pan troglodytes* | Tanzania |
|  | AB821022.1 | *O. stephanostomum* | *Gorilla gorila gorilla* | Gabon |
|  | AB821014.1 | *O. stephanostomum* | *Gorilla gorila gorilla* | Gabon |
| 7. | **LC063699.1** | *O. stephanostomum* | *P. t. schweinfurthii* | Uganda |
| 8. | **LC063706.1** | *O. stephanostomum* | *P. t. schweinfurthii* | Uganda |
| 9. | **LC063708.1** | *O. stephanostomum* | *P. t. schweinfurthii* | Uganda |
| 10. | **AB821015.1** | *O. stephanostomum* | *Gorilla gorila gorilla* | Gabon |
| 11. | **AB821016.1** | *O. stephanostomum* | *Gorilla gorila gorilla* | Gabon |
| 12. | **AB821025.1** | *O. stephanostomum* | *Pan troglodytes* | Gabon |
| 13. | **KR149646.1** | *O. stephanostomum* | *P. t. schweinfurthii* | Uganda |
|  | AB821013.1 | *O. stephanostomum* | *Gorilla gorila gorilla* | Gabon |
|  | AB821017.1 | *O. stephanostomum* | *Pan troglodytes* | Gabon |
|  | AB821018.1 | *O. stephanostomum* | *Gorilla gorila gorilla* | Gabon |
|  | AB821019.1 | *O. stephanostomum* | *Gorilla gorila gorilla* | Gabon |
|  | AB821020.1 | *O. stephanostomum* | *Gorilla gorila gorilla* | Gabon |
|  | AB821021.1 | *O. stephanostomum* | *Gorilla gorila gorilla* | Gabon |
|  | AB821023.1 | *O. stephanostomum* | *Gorilla gorila gorilla* | Gabon |
|  | AB821024.1 | *O. stephanostomum* | *Gorilla gorila gorilla* | Gabon |
|  | AB821026.1 | *O. stephanostomum* | *Pan troglodytes* | Gabon |
|  | AB821027.1 | *O. stephanostomum* | *Gorilla gorila gorilla* | Gabon |
|  | AB821028.1 | *O. stephanostomum* | *Gorilla gorila gorilla* | Gabon |
|  | AB821029.1 | *O. stephanostomum* | *Gorilla gorila gorilla* | Gabon |
|  | AB821030.1 | *O. stephanostomum* | *Gorilla gorila gorilla* | Gabon |
|  | AB821031.1 | *O. stephanostomum* | *Gorilla gorila gorilla* | Gabon |
|  | KR149647.1 | *O. stephanostomum* | *Homo sapiens* | Uganda |
|  | KT592235.1 | *O. stephanostomum* | *P. t. schweinfurthii* | Uganda |
|  | LC063697.1 | *O. stephanostomum* | *P. t. schweinfurthii* | Uganda |
|  | LC063698.1 | *O. stephanostomum* | *P. t. schweinfurthii* | Uganda |
|  | LC063700.1 | *O. stephanostomum* | *P. t. schweinfurthii* | Uganda |
|  | LC063701.1 | *O. stephanostomum* | *P. t. schweinfurthii* | Uganda |
|  | LC063702.1 | *O. stephanostomum* | *P. t. schweinfurthii* | Uganda |
|  | LC063703.1 | *O. stephanostomum* | *P. t. schweinfurthii* | Uganda |
|  | LC063704.1 | *O. stephanostomum* | *P. t. schweinfurthii* | Uganda |
|  | LC063705.1 | *O. stephanostomum* | *P. t. schweinfurthii* | Uganda |
|  | LC063707.1 | *O. stephanostomum* | *P. t. schweinfurthii* | Uganda |
|  | LC063709.1 | *O. stephanostomum* | *P. t. schweinfurthii* | Uganda |
|  | LC063710.1 | *O. stephanostomum* | *P. t. schweinfurthii* | Uganda |
|  | LC063711.1 | *O. stephanostomum* | *P. t. schweinfurthii* | Uganda |
| 14. | **KR149648.1** | *O. stephanostomum* | *P. t. schweinfurthii* | Uganda |
| 15. | **AF136575.1** | *O. bifurcum* | *Cercopithecus mona* | Ghana |
| 16. | **AF136576.1** | *O. stephanostomum* | *Pan troglodytes* | Tanzania |
| 17. | **KF250649.1** | *O. bifurcum* | *Cercopithecus lhoesti* | Uganda |
|  | KF250589.1 | *O. bifurcum* | Red colobus | Uganda |
| 18. | **KF250654.1** | *O. bifurcum* | *Papio anubis* | Uganda |
|  | KF250626.1 | *O. bifurcum* | Red colobus | Uganda |
| 19. | **KF319025.1** | *O. bifurcum* | *Macaca fascicularis* | China |
|  | KF319024.1 | *O. bifurcum* | *Macaca fascicularis* | China |
| 20. | **KF319026.1** | *O. bifurcum* | *Macaca fascicularis* | China |
| 21. | **KR149652.1** | *O. bifurcum* | *Papio anubis* | Uganda |
| 22. | **KR149653.1** | *O. bifurcum* | *P. t. schweinfurthii* | Uganda |
| 23. | **KR149655.1** | *O. bifurcum* | *Papio anubis* | Uganda |
| 24. | **KR149656.1** | *O. bifurcum* | *P. t. schweinfurthii* | Uganda |
| 25. | **KT215379.1** | *O. bifurcum* | *Papio cynocephalus* | Kenya |
| 26. | **LC063694.1** | *O. bifurcum* | *P. t. schweinfurthii* | Uganda |
| 27. | **LC063695.1** | *O. bifurcum* | *P. t. schweinfurthii* | Uganda |
| 28. | **LC063696.1** | *O. bifurcum* | *P. t. schweinfurthii* | Uganda |
|  | KF250587.1 | *O. bifurcum* | *Cercopithecus ascanius* | Uganda |
|  | KF250590.1 | *O. bifurcum* | Red colobus | Uganda |
|  | KF250591.1 | *O. bifurcum* | *Papio anubis* | Uganda |
|  | KF250596.1 | *O. bifurcum* | *Lophocebus albigena* | Uganda |
|  | KF250600.1 | *O. bifurcum* | *Papio anubis* | Uganda |
|  | KF250601.1 | *O. bifurcum* | *Papio anubis* | Uganda |
|  | KF250602.1 | *O. bifurcum* | *Papio anubis* | Uganda |
|  | KF250603.1 | *O. bifurcum* | *Papio anubis* | Uganda |
|  | KF250604.1 | *O. bifurcum* | *Papio anubis* | Uganda |
|  | KF250609.1 | *O. bifurcum* | *Papio anubis* | Uganda |
|  | KF250610.1 | *O. bifurcum* | *Papio anubis* | Uganda |
|  | KF250627.1 | *O. bifurcum* | *Papio anubis* | Uganda |
|  | KF250628.1 | *O. bifurcum* | *Papio anubis* | Uganda |
|  | KF250629.1 | *O. bifurcum* | *Papio anubis* | Uganda |
|  | KR149654.1 | *O. bifurcum* | *Colobus guereza* | Uganda |
|  | KR149657.1 | *O. bifurcum* | *Papio anubis* | Uganda |
|  | KT592234.1 | *O. bifurcum* | *P. t. schweinfurthii* | Uganda |
|  | LC063714.1 | *O. bifurcum* | *Papio cynocephalus* | Tanzania |
|  | LC063715.1 | *O. bifurcum* | *Papio cynocephalus* | Tanzania |
|  | LC063718.1 | *O. bifurcum* | *Papio cynocephalus* | Tanzania |
|  | LC063719.1 | *O. bifurcum* | *Papio ursinus* | SAR |
| 29. | **LC063712.1** | *O. bifurcum* | *Papio cynocephalus* | Tanzania |
| 30. | **LC063713.1** | *O. bifurcum* | *Papio cynocephalus* | Tanzania |
| 31. | **LC063716.1** | *O. bifurcum* | *Papio cynocephalus* | Tanzania |
| 32. | **LC063717.1** | *O. bifurcum* | *Papio cynocephalus* | Tanzania |
|  | LC063693.1 | *O. bifurcum* | *P. t. schweinfurthii* | Uganda |
| 33. | **LC063720.1** | *O. bifurcum* | *Papio ursinus* | SAR |
| 34. | **Y11733.1** | *O. bifurcum* | *Homo sapiens* | Togo |
| 35. | **KF250645.1** | *Oesophagostomum sp.* | *Cercopithecus lhoesti* | Uganda |
|  | KF250646.1 | *Oesophagostomum sp.* | *Cercopithecus lhoesti* | Uganda |
|  | KF250650.1 | *Oesophagostomum sp.* | *Cercopithecus lhoesti* | Uganda |
|  | KF250651.1 | *Oesophagostomum sp.* | *Cercopithecus lhoesti* | Uganda |
|  | KF250652.1 | *Oesophagostomum sp.* | *Cercopithecus lhoesti* | Uganda |
| 36. | **KF250655.1** | *Oesophagostomum sp.* | *Homo sapiens* | Uganda |
|  | KF250657.1 | *Oesophagostomum sp.* | *Homo sapiens* | Uganda |
| 37. | **KF250593.1** | *Oesophagostomum sp.* | *Cercopithecus ascanius* | Uganda |
|  | KF250597.1 | *Oesophagostomum sp.* | *Lophocebus albigena* | Uganda |
|  | KF250607.1 | *Oesophagostomum sp.* | *Lophocebus albigena* | Uganda |
|  | KF250608.1 | *Oesophagostomum sp.* | *Lophocebus albigena* | Uganda |
|  | KF250611.1 | *Oesophagostomum sp.* | *Colobus guereza* | Uganda |
|  | KF250620.1 | *Oesophagostomum sp.* | *Colobus guereza* | Uganda |
|  | KF250621.1 | *Oesophagostomum sp.* | *Colobus guereza* | Uganda |
|  | KF250622.1 | *Oesophagostomum sp.* | *Cercopithecus mitis* | Uganda |
|  | KF250623.1 | *Oesophagostomum sp.* | *Cercopithecus mitis* | Uganda |
|  | KF250624.1 | *Oesophagostomum sp.* | Red colobus | Uganda |
|  | KF250625.1 | *Oesophagostomum sp.* | Red colobus | Uganda |
|  | KF250630.1 | *Oesophagostomum sp.* | *Cercopithecus mitis* | Uganda |
|  | KF250632.1 | *Oesophagostomum sp.* | *Lophocebus albigena* | Uganda |
|  | KF250633.1 | *Oesophagostomum sp.* | *Lophocebus albigena* | Uganda |
|  | KF250636.1 | *Oesophagostomum sp.* | *Cercopithecus ascanius* | Uganda |
|  | KF250656.1 | *Oesophagostomum sp.* | *Homo sapiens* | Uganda |
|  | KF250658.1 | *Oesophagostomum sp.* | *Homo sapiens* | Uganda |
|  | KF250659.1 | *Oesophagostomum sp.* | *Homo sapiens* | Uganda |
|  | KF250660.1 | *Oesophagostomum sp.* | *Homo sapiens* | Uganda |
|  | KR149658.1 | *Oesophagostomum sp.* | *Papio anubis* | Uganda |
| 38. | **KF319023.1** | *O. aculeatum* | *Macaca fascicularis* | China |
|  | KF319022.1 | *O. aculeatum* | *Macaca fascicularis* | China |
| 39. | **LC063721.1** | *O. aculeatum* | *Macaca fuscata* | Japan |
| 40. | **LC063722.1** | *O. aculeatum* | *Macaca fuscata* | Japan |
| 41. | **LC428824.1*** | *O. aculeatum* | *Pongo pygmaeus* | Malaysia |

SAR – South African Republic

* - Even though the sequence is labeled as *O. aculeatum*, it is probably *Ternidens deminutus* and is not included in the final tree

**Table S2-B**

List of *Oesophagostumum* spp*.* ITS2 sequences generated in this study, their assignment to the haplogroups, and Genbank accesion numbers together with the information on host and locality. The sequences in bold are representatives for the given haplogroup and were used in the phylogenetic tree.

| **Haplotype** | **Sequence** | **Accession number** | **Host** | **Locality** |
| --- | --- | --- | --- | --- |
| **OH1 (N=107)** | **913_CHI_GAB** | MW528465 | *Pan troglodytes* | Gabon, MKT |
|  | 762_CHI_CAM |  | *Pan troglodytes* | Cameroon, MB |
|  | 769_CHI_ CAM |  | *Pan troglodytes* | Cameroon, MB |
|  | 774_CHI_ CAM |  | *Pan troglodytes* | Cameroon, MB |
|  | 775_CHI_ CAM |  | *Pan troglodytes* | Cameroon, MB |
|  | 914_CHI_GAB |  | *Pan troglodytes* | Gabon, MKT |
|  | 917_CHI_GAB |  | *Pan troglodytes* | Gabon, MKT |
|  | 932_CHI_GAB |  | *Pan troglodytes* | Gabon, MKT |
|  | 934_CHI_GAB |  | *Pan troglodytes* | Gabon, MKT |
|  | 941_CHI_GAB |  | *Pan troglodytes* | Gabon, MKT |
|  | 949_CHI_GAB |  | *Pan troglodytes* | Gabon, MKT |
|  | 951_CHI_GAB |  | *Pan troglodytes* | Gabon, MKT |
|  | 953_CHI_GAB |  | *Pan troglodytes* | Gabon, MKT |
|  | 956_CHI_GAB |  | *Pan troglodytes* | Gabon, MKT |
|  | 956_CHI_CAM |  | *Pan troglodytes* | Cameroon, SOM |
|  | 957_CHI_GAB |  | *Pan troglodytes* | Gabon, MKT |
|  | 960_CHI_CAM |  | *Pan troglodytes* | Cameroon, SOM |
|  | 962_CHI_CAM |  | *Pan troglodytes* | Cameroon, SOM |
|  | 977_CHI_GAB |  | *Pan troglodytes* | Gabon, MKT |
|  | 987_CHI_GAB |  | *Pan troglodytes* | Gabon, MKT |
|  | 988_CHI_GAB |  | *Pan troglodytes* | Gabon, MKT |
|  | 990_CHI_CAM |  | *Pan troglodytes* | Cameroon, SOM |
|  | 991_CHI_GAB |  | *Pan troglodytes* | Gabon, MKT |
|  | 994_CHI_GAB |  | *Pan troglodytes* | Gabon, MKT |
|  | 995_CHI_GAB |  | *Pan troglodytes* | Gabon, MKT |
|  | 998_CHI_GAB |  | *Pan troglodytes* | Gabon, MKT |
|  | 999_CHI_GAB |  | *Pan troglodytes* | Gabon, MKT |
|  | 1000_CHI_GAB |  | *Pan troglodytes* | Gabon, MKT |
|  | 1002_CHI_GAB |  | *Pan troglodytes* | Gabon, MKT |
|  | 1008_CHI_GAB |  | *Pan troglodytes* | Gabon, MKT |
|  | 1015_CHI_GAB |  | *Pan troglodytes* | Gabon, MKT |
|  | 1022_CHI_GAB |  | *Pan troglodytes* | Gabon, MKT |
|  | 1023_CHI_GAB |  | *Pan troglodytes* | Gabon, MKT |
|  | 1024_CHI_GAB_B |  | *Pan troglodytes* | Gabon, MKT |
|  | 1028_CHI_GAB |  | *Pan troglodytes* | Gabon, MKT |
|  | 1029_CHI_GAB |  | *Pan troglodytes* | Gabon, MKT |
|  | 1032_CHI_GAB |  | *Pan troglodytes* | Gabon, MKT |
|  | 1169_CHI_GAB |  | *Pan troglodytes* | Gabon, MKT |
|  | 1179_CHI_GAB |  | *Pan troglodytes* | Gabon, MKT |
|  | 1188_CHI_GAB |  | *Pan troglodytes* | Gabon, MKT |
|  | 1225_CHI_GAB |  | *Pan troglodytes* | Gabon, MKT |
|  | 1226_CHI_GAB |  | *Pan troglodytes* | Gabon, MKT |
|  | 1228_CHI_GAB |  | *Pan troglodytes* | Gabon, MKT |
|  | 1236_CHI_GAB |  | *Pan troglodytes* | Gabon, MKT |
|  | 1243_CHI_GAB_B |  | *Pan troglodytes* | Gabon, MKT |
|  | 1250_CHI_GAB |  | *Pan troglodytes* | Gabon, MKT |
|  | 1264_CHI_GAB |  | *Pan troglodytes* | Gabon, MKT |
|  | 1284_CHI_GAB_A |  | *Pan troglodytes* | Gabon, MKT |
|  | 1294_CHI_GAB |  | *Pan troglodytes* | Gabon, MKT |
|  | 1295_CHI_GAB |  | *Pan troglodytes* | Gabon, MKT |
|  | 1298_CHI_GAB |  | *Pan troglodytes* | Gabon, MKT |
|  | 1299_CHI_GAB |  | *Pan troglodytes* | Gabon, MKT |
|  | 1316_CHI_GAB |  | *Pan troglodytes* | Gabon, MKT |
|  | 1330_CHI_GAB |  | *Pan troglodytes* | Gabon, MKT |
|  | 1331_CHI_GAB_B |  | *Pan troglodytes* | Gabon, MKT |
|  | 1340_CHI_GAB |  | *Pan troglodytes* | Gabon, MKT |
|  | 1344_CHI_GAB |  | *Pan troglodytes* | Gabon, MKT |
|  | 1345_CHI_GAB |  | *Pan troglodytes* | Gabon, MKT |
|  | 1347_CHI_GAB |  | *Pan troglodytes* | Gabon, MKT |
|  | 1369_CHI_GAB |  | *Pan troglodytes* | Gabon, MKT |
|  | 1464_CHI_GAB |  | *Pan troglodytes* | Gabon, MKT |
|  | 1468_CHI_GAB_A |  | *Pan troglodytes* | Gabon, MKT |
|  | 1472_CHI_GAB |  | *Pan troglodytes* | Gabon, MKT |
|  | 1508_CHI_GAB |  | *Pan troglodytes* | Gabon, MKT |
|  | 1972_CHI_GAB |  | *Pan troglodytes* | Gabon, MKT |
|  | 2307_CHI_GAB |  | *Pan troglodytes* | Gabon, MKT |
|  | 2313_CHI_GAB |  | *Pan troglodytes* | Gabon, MKT |
|  | 2347_CHI_GAB |  | *Pan troglodytes* | Gabon, MKT |
|  | 2480_CHI_GAB |  | *Pan troglodytes* | Gabon, MKT |
|  | 2492_CHI_GAB_A |  | *Pan troglodytes* | Gabon, MKT |
|  | 2504_CHI_GAB |  | *Pan troglodytes* | Gabon, MKT |
|  | 5149_AGILIS_CAM_A | MW621352 | *Cercocebus agilis* | Cameroon, MB |
|  | 5150_AGILIS_CAM_A |  | *Cercocebus agilis* | Cameroon, MB |
|  | 4874_CHI_CAM | MW621348 | *Pan troglodytes* | Cameroon, SOM |
|  | 4875_CHI_CAM |  | *Pan troglodytes* | Cameroon, SOM |
|  | 4876_CHI_CAM |  | *Pan troglodytes* | Cameroon, SOM |
|  | 4877_CHI_CAM |  | *Pan troglodytes* | Cameroon, SOM |
|  | 6945_CHI_CAM_A |  | *Pan troglodytes* | Cameroon, MB |
|  | 6948_CHI_CAM | MW621355 | *Pan troglodytes* | Cameroon, MB |
|  | 6950_CHI_CAM |  | *Pan troglodytes* | Cameroon, MB |
|  | 6951_CHI_CAM |  | *Pan troglodytes* | Cameroon, MB |
|  | 6952_CHI_CAM |  | *Pan troglodytes* | Cameroon, MB |
|  | 6955_CHI_CAM |  | *Pan troglodytes* | Cameroon, MB |
|  | 6956_CHI_CAM |  | *Pan troglodytes* | Cameroon, MB |
|  | 6957_CHI_CAM |  | *Pan troglodytes* | Cameroon, MB |
|  | 6958_CHI_CAM_A |  | *Pan troglodytes* | Cameroon, MB |
|  | 6959_CHI_CAM_A |  | *Pan troglodytes* | Cameroon, MB |
|  | 8385_CHI_CAM_A |  | *Pan troglodytes* | Cameroon, MB |
|  | 5324_GOR_CAM_A |  | *Gorilla gorilla* | Cameroon, MB |
|  | 5333_GOR_CAM_A |  | *Gorilla gorilla* | Cameroon, MB |
|  | 5360_GOR_CAM_A |  | *Gorilla gorilla* | Cameroon, MB |
|  | 9818_GOR_CAM | MW621358 | *Gorilla gorilla* | Cameroon, SOM |
|  | 9819_GOR_CAM |  | *Gorilla gorilla* | Cameroon, SOM |
|  | 9820_GOR_CAM |  | *Gorilla gorilla* | Cameroon, SOM |
|  | 9821_GOR_CAM |  | *Gorilla gorilla* | Cameroon, SOM |
|  | 9822_GOR_CAM |  | *Gorilla gorilla* | Cameroon, SOM |
|  | 9823_GOR_CAM |  | *Gorilla gorilla* | Cameroon, SOM |
|  | 9824_GOR_CAM |  | *Gorilla gorilla* | Cameroon, SOM |
|  | 9826_GOR_CAM |  | *Gorilla gorilla* | Cameroon, SOM |
|  | 9829_GOR_CAM |  | *Gorilla gorilla* | Cameroon, SOM |
|  | 9830_GOR_CAM |  | *Gorilla gorilla* | Cameroon, SOM |
|  | 9846_GOR_CAM |  | *Gorilla gorilla* | Cameroon, SOM |
|  | 9847_GOR_CAM |  | *Gorilla gorilla* | Cameroon, SOM |
|  | 9848_GOR_CAM |  | *Gorilla gorilla* | Cameroon, SOM |
|  | 9965_GOR_CAM | MW621361 | *Gorilla gorilla* | Cameroon, LB |
|  | 10015_GOR_CAM_A | MW621363 | *Gorilla gorilla* | Cameroon, MB |
|  | 10017_GOR_CAM_A |  | *Gorilla gorilla* | Cameroon, MB |
| **OH2 (N=33)** | **962_CHI_GAB** | MW528472 | *Pan troglodytes* | Gabon, MKT |
|  | 989_CHI_GAB |  | *Pan troglodytes* | Gabon, MKT |
|  | 988_CHI_CAM_R_B |  | *Pan troglodytes* | Cameroon, SOM |
|  | 989_CHI_CAM_R_A | MW621343 | *Pan troglodytes* | Cameroon, SOM |
|  | 1003_CHI_GAB |  | *Pan troglodytes* | Gabon, MKT |
|  | 1011_CHI_GAB |  | *Pan troglodytes* | Gabon, MKT |
|  | 1214_CHI_GAB |  | *Pan troglodytes* | Gabon, MKT |
|  | 1267_CHI_GAB |  | *Pan troglodytes* | Gabon, MKT |
|  | 1277_CHI_GAB |  | *Pan troglodytes* | Gabon, MKT |
|  | 1279_CHI_GAB |  | *Pan troglodytes* | Gabon, MKT |
|  | 1296_CHI_GAB |  | *Pan troglodytes* | Gabon, MKT |
|  | 1301_CHI_GAB_B |  | *Pan troglodytes* | Gabon, MKT |
|  | 1305_CHI_GAB_B |  | *Pan troglodytes* | Gabon, MKT |
|  | 1334_CHI_GAB |  | *Pan troglodytes* | Gabon, MKT |
|  | 1342_CHI_GAB |  | *Pan troglodytes* | Gabon, MKT |
|  | 2364_CHI_GAB |  | *Pan troglodytes* | Gabon, MKT |
|  | 2366_CHI_GAB |  | *Pan troglodytes* | Gabon, MKT |
|  | 2501_CHI_GAB |  | *Pan troglodytes* | Gabon, MKT |
|  | 5134_CHI_CAM_B |  | *Pan troglodytes* | Cameroon, MB |
|  | 5136_CHI_CAM_B |  | *Pan troglodytes* | Cameroon, MB |
|  | 5137_CHI_GAB |  | *Pan troglodytes* | Gabon, MKT |
|  | 5138_CHI_CAM | MW621350 | *Pan troglodytes* | Cameroon, MB |
|  | 5147_CHI_GAB_B |  | *Pan troglodytes* | Gabon, MKT |
|  | 5151_CHI_CAM_B |  | *Pan troglodytes* | Cameroon, MB |
|  | 5157_CHI_CAM_B |  | *Pan troglodytes* | Cameroon, MB |
|  | 5354_GOR_CAM |  | *Gorilla gorilla* | Cameroon, MB |
|  | 9805_GOR_CAM_A |  | *Gorilla gorilla* | Cameroon, SOM |
|  | 9827_GOR_CAM | MW621359 | *Gorilla gorilla* | Cameroon, SOM |
|  | 9835_GOR_CAM |  | *Gorilla gorilla* | Cameroon, SOM |
|  | 9838_GOR_CAM_R_A |  | *Gorilla gorilla* | Cameroon, SOM |
|  | 9844_GOR_CAM_R_B |  | *Gorilla gorilla* | Cameroon, SOM |
|  | 10013_GOR_CAM | MW621362 | *Gorilla gorilla* | Cameroon, MB |
| **OH1 or OH2 (N=13)** | 944_CHI_GAB |  | *Pan troglodytes* | Gabon, MKT |
|  | 950_CHI_GAB |  | *Pan troglodytes* | Gabon, MKT |
|  | 965_CHI_CAM |  | *Pan troglodytes* | Cameroon, SOM |
|  | 966_CHI_CAM |  | *Pan troglodytes* | Cameroon, SOM |
|  | 984_CHI_CAM |  | *Pan troglodytes* | Cameroon, SOM |
|  | 1266_CHI_GAB |  | *Pan troglodytes* | Gabon, MKT |
|  | 1291_CHI_GAB |  | *Pan troglodytes* | Gabon, MKT |
|  | 1311_CHI_GAB |  | *Pan troglodytes* | Gabon, MKT |
|  | 1350_CHI_GAB |  | *Pan troglodytes* | Gabon, MKT |
|  | 5338_GOR_CAM |  | *Gorilla gorilla* | Cameroon, MB |
|  | 9801_GOR_CAM |  | *Gorilla gorilla* | Cameroon, SOM |
|  | 9802_GOR_CAM |  | *Gorilla gorilla* | Cameroon, SOM |
|  | 9825_GOR_CAM |  | *Gorilla gorilla* | Cameroon, SOM |
| **OH3 (N=20)** | **1024_CHI_GAB_A** | MW528473 | *Pan troglodytes* | Gabon, MKT |
|  | 1220_CHI_GAB |  | *Pan troglodytes* | Gabon, MKT |
|  | 1243_CHI_GAB_A |  | *Pan troglodytes* | Gabon, MKT |
|  | 1284_CHI_GAB_B |  | *Pan troglodytes* | Gabon, MKT |
|  | 1331_CHI_GAB_A |  | *Pan troglodytes* | Gabon, MKT |
|  | 1468_CHI_GAB_B |  | *Pan troglodytes* | Gabon, MKT |
|  | 2492_CHI_GAB_B |  | *Pan troglodytes* | Gabon, MKT |
|  | 5149_AGILIS_CAM_B | MW621353 | *Cercocebus agilis* | Cameroon, MB |
|  | 5150_AGILIS_CAM_B |  | *Cercocebus agilis* | Cameroon, MB |
|  | 6945_CHI_CAM_B | MW621354 | *Pan troglodytes* | Cameroon, MB |
|  | 6958_CHI_CAM_B |  | *Pan troglodytes* | Cameroon, MB |
|  | 6959_CHI_CAM_B |  | *Pan troglodytes* | Cameroon, MB |
|  | 8385_CHI_CAM_B |  | *Pan troglodytes* | Cameroon, MB |
|  | 5324_GOR_CAM_B |  | *Gorilla gorilla* | Cameroon, MB |
|  | 5333_GOR_CAM_B |  | *Gorilla gorilla* | Cameroon, MB |
|  | 5360_GOR_CAM_B |  | *Gorilla gorilla* | Cameroon, MB |
|  | 9845_GOR_CAM | MW621360 | *Gorilla gorilla* | Cameroon, SOM |
|  | 9849_GOR_CAM |  | *Gorilla gorilla* | Cameroon, SOM |
|  | 10015_GOR_CAM_B | MW621364 | *Gorilla gorilla* | Cameroon, MB |
|  | 10017_GOR_CAM_B |  | *Gorilla gorilla* | Cameroon, MB |
| **OH4 (N=9)** | **1301_CHI_GAB_A** | MW528474 | *Pan troglodytes* | Gabon, MKT |
|  | 1000_CHI_CAM_R_B | MW621345 | *Pan troglodytes* | Cameroon, SOM |
|  | 1305_CHI_GAB_A |  | *Pan troglodytes* | Gabon, MKT |
|  | 5134_CHI_CAM_A | MW621349 | *Pan troglodytes* | Cameroon, MB |
|  | 5136_CHI_CAM_A |  | *Pan troglodytes* | Cameroon, MB |
|  | 5147_CHI_GAB_A |  | *Pan troglodytes* | Gabon, MKT |
|  | 5148_CHI_CAM_A |  | *Pan troglodytes* | Cameroon, MB |
|  | 5151_CHI_CAM_A |  | *Pan troglodytes* | Cameroon, MB |
|  | 5157_CHI_CAM_A |  | *Pan troglodytes* | Cameroon, MB |
| **OH5 (N=7)** | **939_CHI_GAB_A** | MW528466 | *Pan troglodytes* | Gabon, MKT |
|  | 988_CHI_CAM_R_A | MW621344 | *Pan troglodytes* | Cameroon, SOM |
|  | 989_CHI_CAM_R_B |  | *Pan troglodytes* | Cameroon, SOM |
|  | 1000_CHI_CAM_R_A |  | *Pan troglodytes* | Cameroon, SOM |
|  | 5148_CHI_CAM_B | MW621351 | *Pan troglodytes* | Cameroon, MB |
|  | 9805_GOR_CAM_B | MW621357 | *Gorilla gorilla* | Cameroon, SOM |
|  | 9838_GOR_CAM_R_B |  | *Gorilla gorilla* | Cameroon, SOM |
| **OH6 (N=1)** | **954_CHI_GAB** | MW528468 | *Pan troglodytes* | Gabon, MKT |
| **OH7 (N=1)** | **9844_GOR_CAM_R_A** | MW528480 | *Gorilla gorilla* | Cameroon, SOM |
| **OH8 (N=3)** | **9803_GOR_CAM** | MW528479 | *Gorilla gorilla* | Cameroon, SOM |
|  | 9804_GOR_CAM |  | *Gorilla gorilla* | Cameroon, SOM |
|  | 9806_GOR_CAM |  | *Gorilla gorilla* | Cameroon, SOM |
| **OH9 (N= 4)** | **957_CHI_CAM_B** | MW528470 | *Pan troglodytes* | Cameroon, SOM |
|  | 961_CHI_CAM_A |  | *Pan troglodytes* | Cameroon, SOM |
|  | 3342_CEB_CAM_A | MW621347 | *Cercocebus torquatus* | Cameroon, DJ |
|  | 6954_CHI_CAM | MW621356 | *Pan troglodytes* | Cameroon, MB |
| **OH10 (N=2)** | **957_CHI_CAM_A** | MW528469 | *Pan troglodytes* | Cameroon, SOM |
|  | 958_CHI_CAM_A |  | *Pan troglodytes* | Cameroon, SOM |
| **OH11 (N=2)** | **958_CHI_CAM_B** | MW528471 | *Pan troglodytes* | Cameroon, SOM |
|  | 961_CHI_CAM_B |  | *Pan troglodytes* | Cameroon, SOM |
| **OH12 (N=1)** | **3342_CEB_CAM_B** | MW528476 | *Cercocebus torquatus* | Cameroon, DJ |
| **OH13 (N=2)** | **9800_CEB_CAM_A** | MW528477 | *Cercocebus torquatus* | Cameroon, SOM |
|  | 3341_CEB_CAM | MW621346 | *Cercocebus torquatus* | Cameroon, DJ |
| **OH14 (N=1)** | **9800_CEB_CAM_B** | MW528478 | *Cercocebus torquatus* | Cameroon, SOM |
| **OH15 (N=3)** | **3335_MDR_CAM** | MW528475 | *Mandrillus sphinx* | Cameroon, DJ |
|  | 3336_MDR_CAM |  | *Mandrillus sphinx* | Cameroon, DJ |
|  | 3337_MDR_CAM |  | *Mandrillus sphinx* | Cameroon, DJ |
| **OH16 (N=1)** | **939_CHI_GAB_B** | MW528467 | *Pan troglodytes* | Gabon, MKT |
| **OH17(N=4)*** | 8386_CHI_CAM | MW617066 | *Pan troglodytes* | Cameroon, MB |
|  | 4879_CRP_CAM | MW617064 | *Cercopithecus nictitans* | Cameroon, SOM |
|  | 4880_CRP_CAM | MW617064 | *Cercopithecus nictitans* | Cameroon, SOM |
|  | 6960_CRP_CAM | MW617065 | *Cercopithecus nictitans* | Cameroon, MB |

DJ – Djoum; MB – Mambele NP; MKT – Makamangoye; LB – Lobéké NP; SOM – Somalomo

* - This haplotypes belong to *Ternidens deminutus* and this haplotype was not used in the phylogenetic tree

**Table S3** Sequences of *Trichuris* spp. Downloaded from GenBank and used in phylogenetic analysis

| Hap | Accession No. | Name | Host | Country |
| --- | --- | --- | --- | --- |
| **1** | **GQ301554** | *T. trichiura* | *Papio hamadryas* | South Africa |
| **2** | **KP336482** | *Trichuris* sp. | *Chlorocebus aethiops* | Italy |
| **3** | **KJ588160** | *T. trichiura* | *Pan troglodytes* | Uganda |
| **4** | **KP336479** | *Trichuris* sp. | *Chlorocebus aethiops* | Italy |
| **5** | **KP336484** | *Trichuris* sp. | *Chlorocebus aethiops* | Italy |
|  | KP336480 | *Trichuris* sp. | *Chlorocebus aethiops* | Italy |
|  | KP336478 | *Trichuris* sp. | *Chlorocebus aethiops* | Italy |
|  | KP336483 | *Trichuris* sp. | *Chlorocebus aethiops* | Italy |
|  | KP336481 | *Trichuris* sp. | *Chlorocebus aethiops* | Italy |
|  | KJ588153 | *T. trichiura* | *Cercopithecus ascanius* | Uganda |
|  | KJ588165 | *T. trichiura* | *Cercopithecus mitis* | Uganda |
|  | KJ588149 | *T. trichiura* | *Colobus guereza* | Uganda |
|  | KJ588148 | *T. trichiura* | *Cercopithecus ascanius* | Uganda |
|  | KJ588139 | *T. trichiura* | *Colobus guereza* | Uganda |
|  | KJ588146 | *T. trichiura* | *Colobus guereza* | Uganda |
|  | KJ588144 | *T. trichiura* | *Procolobus rufonitratus* | Uganda |
|  | KJ588138 | *T. trichiura* | *Cercopithecus mitis* | Uganda |
| **6** | **KJ588142** | *T. trichiura* | *Procolobus rufonitratus* | Uganda |
|  | KJ588166 | *T. trichiura* | *Colobus guereza* | Uganda |
|  | KJ588141 | *T. trichiura* | *Cercopithecus mitis* | Uganda |
|  |  |  |  |  |
| **7** | **KJ588156** | *T. trichiura* | *Cercopithecus mitis* | Uganda |
| **8** | **GQ301555** | *T. trichiura* | *Homo sapiens* | Cameroon |
| **9** | **KJ588162** | *T. trichiura* | *Procolobus rufonitratus* | Uganda |
| **10** | **KJ588137** | *T. trichiura* | *Procolobus rufonitratus* | Uganda |
| **11** | **JF690944** | *Trichuris* sp. | *Chlorocebus aethiops salaeus* | Czech Republic |
|  | JF690945 | *Trichuris* sp. | *Macaca silenus* | Czech Republic |
|  | KJ588135 | *T. trichiura* | *Cercopithecus ascanius* | Uganda |
|  | KJ588157 | *T. trichiura* | *Colobus guereza* | Uganda |
|  | KJ588158 | *T. trichiura* | *Colobus guereza* | Uganda |
|  | KJ588159 | *T. trichiura* | *Homo sapiens* | Uganda |
|  | KJ588161 | *T. trichiura* | *Colobus guereza* | Uganda |
|  | KJ588164 | *T. trichiura* | *Cercopithecus lhoesti* | Uganda |
|  | KJ588163 | *T. trichiura* | *Cercopithecus lhoesti* | Uganda |
|  | KJ588140 | *T. trichiura* | *Procolobus rufonitratus* | Uganda |
|  | KJ588150 | *T. trichiura* | *Cercopithecus ascanius* | Uganda |
|  | KJ588145 | *T. trichiura* | *Cercopithecus ascanius* | Uganda |
|  | KJ588152 | *T. trichiura* | *Papio anubis* | Uganda |
|  | KJ588154 | *T. trichiura* | *Papio anubis* | Uganda |
|  | KJ588147 | *T. trichiura* | *Lophocebus albigena* | Uganda |
|  | KJ588143 | *T. trichiura* | *Cercopithecus mitis* | Uganda |
|  | KJ588155 | *T. trichiura* | *Papio anubis* | Uganda |
|  | KJ588151 | *T. trichiura* | *Homo sapiens* | Uganda |
|  | KJ588136 | *T. trichiura* | *Colobus guereza* | Uganda |
| **12** | **FM991955** | *T. colobae* | *Colobus guereza kikuyuensis* | Spain |
|  | FM991956 | *T. colobae* | *Nomascus gabriellae* | Spain |
| **13** | **AM993002** | *T. suis* | N/A | China |
| **14** | **AM993012** | *T. suis* | N/A | China |
|  | AM993013 | *T. suis* | N/A | China |
| **15** | **MG656444** | *T. suis* | N/A | China |
| **16** | **JN181822** | *T. trichiura* | *Homo sapiens* | Jamaica |
| **17** | **JN181823** | *T. trichiura* | *Homo sapiens* | Uganda |
| **18** | **AM993007** | *T. suis* | N/A | China |
| **19** | **AM993001** | *T. suis* | N/A | China |
| **20** | **JN181771** | *T. suis* | Pig | Uganda |
| **21** | **JN181830** | *T. trichiura* | *Homo sapiens* | Uganda |
|  | JN181829 | *T. trichiura* | *Homo sapiens* | Uganda |
| **22** | **JN181821** | *T. trichiura* | *Homo sapiens* | Jamaica |
| **23** | **JN181824** | *T. trichiura* | *Homo sapiens* | Uganda |
| **24** | **JN181772** | *T. suis* | Pig | USA |
|  | JN181817 | *T. trichiura* | *Homo sapiens* | Jamaica |
|  | JN181818 | *T. trichiura* | *Homo sapiens* | Jamaica |
|  | JN181819 | *T. trichiura* | *Homo sapiens* | Jamaica |
|  | JN181826 | *T. trichiura* | *Homo sapiens* | Uganda |
| **25** | **JN181815** | *T. trichiura* | *Homo sapiens* | Uganda |
| **26** | **JN181769** | *T. suis* | Pig | Denmark |
|  | JN181814 | *T. trichiura* | *Homo sapiens* | Uganda |
| **27** | **JN181782** | *T. suis* | Pig | Uganda |
| **28** | **JN181784** | *T. suis* | Pig | Uganda |
| **29** | **JN181816** | *T. trichiura* | *Homo sapiens* | Uganda |
| **30** | **JN181792** | *T. suis* | Pig | Uganda |
| **31** | **JN181804** | *T. suis* | Pig | Tanzania |
| **32** | **JN181789** | *T. suis* | Pig | Uganda |
|  | JN181812 | *T. trichiura* | *Homo sapiens* | Uganda |
|  | JN181813 | *T. trichiura* | *Homo sapiens* | Uganda |
| **33** | **JF690943** | *Trichuris* sp. | *Theropithecus gelada* | Czech Republic |
| **34** | **JN181856** | *T. trichiura* | *Homo sapiens* | Uganda |
| **35** | **JF690940** | *Trichuris* sp. | *Homo sapiens* | Czech Republic |
| **36** | **JN181855** | *T. trichiura* | *Homo sapiens* | Uganda |
| **37** | **JN181854** | *T. trichiura* | *Homo sapiens* | Uganda |
|  | JN181852 | *T. trichiura* | *Homo sapiens* | Uganda |
|  | JN181853 | *T. trichiura* | *Homo sapiens* | Uganda |
| **38** | **JN181842** | *T. trichiura* | *Homo sapiens* | Uganda |
|  | JN181841 | *T. trichiura* | *Homo sapiens* | Uganda |
|  | JN181835 | *T. trichiura* | *Homo sapiens* | Uganda |
| **39** | **JN181839** | *T. trichiura* | *Homo sapiens* | Uganda |
| **40** | **JN181836** | *T. trichiura* | *Homo sapiens* | Uganda |
| **41** | **JN181847** | *T. trichiura* | *Homo sapiens* | Uganda |
| **42** | **JN181825** | *T. trichiura* | *Homo sapiens* | Uganda |
| **43** | **JN181831** | *T. trichiura* | *Homo sapiens* | Uganda |
| **44** | **JN181832** | *T. trichiura* | *Homo sapiens* | Uganda |
| **45** | **JN181851** | *T. trichiura* | *Homo sapiens* | Uganda |
| **46** | **JN181820** | *T. trichiura* | *Homo sapiens* | Jamaica |
| **47** | **KF410637** | *Trichuris* sp. | *Papio hamadryas anubis* | USA |
|  | KF410636 | *Trichuris* sp. | *Papio hamadryas* | Denmark |
|  | KF410635 | *Trichuris* sp. | *Papio hamadryas anubis/ Papio h. cynocephalus* | USA |
| **48** | **KX961651** | *Trichuris* sp. | *Chlorocebus aethiops salaeus* | St. Kitt‘s |
| **49** | **JF690941** | *Trichuris* sp. | *Papio hamadryas* | Czech Republic |
|  | JF690950 | *Trichuris* sp. | *Chlorocebus aethiops* | Tanzania |
|  | MH390360 | *Trichuris* sp. | *Papio anubis* | China |
|  | MH390361 | *Trichuris* sp. | *Papio anubis* | China |
|  | MH390359 | *Trichuris* sp. | *Papio anubis* | China |
|  | KT344826 | *Trichuris* sp. | *Papio anubis* | China |
|  | KX961646 | *Trichuris* sp. | *Chlorocebus aethiops salaeus* | St. Kitts and Nevis |
|  | KX961642 | *Trichuris* sp. | *Chlorocebus aethiops salaeus* | St. Kitts and Nevis |
|  | KX961649 | *Trichuris* sp. | *Chlorocebus aethiops salaeus* | St. Kitts and Nevis |
|  | KX961650 | *Trichuris* sp. | *Chlorocebus aethiops salaeus* | St. Kitts and Nevis |
|  | KX961653 | *Trichuris* sp. | *Chlorocebus aethiops salaeus* | St. Kitts and Nevis |
|  | JN181827 | *T. trichiura* | *Homo sapiens* | Uganda |
|  | JN181849 | *T. trichiura* | *Homo sapiens* | Uganda |
|  | JN181850 | *T. trichiura* | *Homo sapiens* | Uganda |
| **50** | **JF690948** | *Trichuris* sp. | *Pan troglodytes* | Netherlands |
| **51** | **JN181838** | *T. trichiura* | *Homo sapiens* | Uganda |
| **52** | **JN181846** | *T. trichiura* | *Homo sapiens* | Uganda |
| **53** | **JN181848** | *T. trichiura* | *Homo sapiens* | Uganda |
| **54** | **JN181859** | *T. trichiura* | *Homo sapiens* | Uganda |
|  | JN181857 | *T. trichiura* | *Homo sapiens* | Uganda |
| **55** | **KF410638** | *Trichuris* sp. | *Papio hamadryas anubis* | USA |
| **56** | **JN181828** | *T. trichiura* | *Homo sapiens* | Uganda |
| **57** | **JN181858** | *T. trichiura* | *Homo sapiens* | Uganda |
| **58** | **MH390364** | *Trichuris* sp. | *Macaca leonina* | China |
|  | MH390365 | *Trichuris* sp. | *Trichuris* sp. | China |
|  | KT344828 | *Trichuris* sp. | *Trichuris* sp. | China |
| **59** | **JF690946** | *Trichuris* sp. | Macaca fascicularis | Czech Republic |
| **60** | **AM992994** | *T. trichiura* | N/A | China |
|  | JN181837 | *T. trichiura* | *Homo sapiens* | Uganda |
| **61** | **AM992996** | *T. trichiura* | N/A | China |
| **62** | **JF690942** | *Trichuris* sp. | *Papio anubis* | Czech Republic |
| **63** | **MN447326** | *Trichuris* sp. | *Macaca mulatta* | N/A |
| **64** | **JN181843** | *T. trichiura* | *Homo sapiens* | Uganda |
| **65** | **JN181860** | *T. trichiura* | *Homo sapiens* | Uganda |
| **66** | **KX961644** | *Trichuris* sp. | *Chlorocebus aethiops salaeus* | St. Kitt‘s |
| **67** | **JN181845** | *T. trichiura* | *Homo sapiens* | Uganda |
| **68** | **JN181840** | *T. trichiura* | *Homo sapiens* | Uganda |
| **69** | **AM992995** | *T. trichiura* | N/A | China |
|  | AM992984 | *T. trichiura* | N/A | China |
| **70** | **KX961647** | *Trichuris* sp. | *Chlorocebus aethiops salaeus* | St. Kitts and Nevis |
| **71** | **KX961652** | *Trichuris* sp. | *Chlorocebus aethiops salaeus* | St. Kitts and Nevis |
| **72** | **MN447328** | *Trichuris* sp. | *Papio anubis* | N/A |
| **73** | **KT344825** | *Trichuris* sp. | *Rhinopithecus roxellana* | China |
| **74** | **JF690949** | *Trichuris* sp. | *Chlorocebus aethiops* | Tanzania |
| **75** | **KT344827** | *Trichuris* sp. | *Chlorocebus aethiops* | China |
|  | MH390370 | *Trichuris* sp. | *Chlorocebus aethiops* | China |
| **76** | **KX961645** | *Trichuris* sp. | *Chlorocebus aethiops salaeus* | St. Kitts and Nevis |
| **77** | **KX961648** | *Trichuris* sp. | *Chlorocebus aethiops salaeus* | St. Kitts and Nevis |
| **78** | **KT344831** | *Trichuris* sp. | *Papio hamadryas* | China |
|  | KT344830 | *Trichuris* sp. | *Nomascus leucogenys* | China |
|  | MH390366 | *Trichuris* sp. | *Nomascus leucogenys* | China |
|  | MH390363 | *Trichuris* sp. | *Papio hamadryas* | China |
| **79** | GQ301553 | *Trichuris* sp. | *Papio hamadryas ursinus* | South Africa |
|  | GQ301552 | *Trichuris* sp. | *Papio hamadryas ursinus* | South Africa |
| **80** | **GQ301551** | *Trichuris* sp. | *Papio hamadryas ursinus* | South Africa |
| **81** | **KX9616643** | *Trichuris* sp. | *Chlorocebus aethiops salaeus* | St. Kitts and Nevis |
| **82** | **KC877992** | *T. trichiura* | *Papio hamadryas* | Turkey |
| **83** | **AM992998** | *T. trichiura* | N/A | China |
| **84** | **AM992997** | *T. trichiura* | N/A | China |
| **85** | **AM992993** | *T. trichiura* | N/A | China |
| **86** | **AM992992** | *T. trichiura* | N/A | China |
|  | AM992990 | *T. trichiura* | N/A | China |
| **87** | **AM992991** | *T. trichiura* | N/A | China |
| **88** | **AM992987** | *T. trichiura* | N/A | China |
|  | AM992988 | *T. trichiura* | N/A | China |
|  | AM992989 | *T. trichiura* | N/A | China |
| **89** | **AM992986** | *T. trichiura* | N/A | China |
|  | AM992985 | *T. trichiura* | N/A | China |
| **90** | **AM992982** | *T. trichiura* | N/A | China |
|  | AM992983 | *T. trichiura* | N/A | China |
|  | AM992981 | *T. trichiura* | N/A | China |
| **91** | **JF690947** | *Trichuris* sp. | *Nomascus gabriellae* | Slovakia |
| **92** | **MN44732** | *Trichuris* sp. | *Trachypithecus francoisi* | China |
| **93** | **KT186233** | *Trichuris* sp. | *Trachypithecus francoisi* | China |
| **94** | **KT186232** | *Trichuris* sp. | *Trachypithecus francoisi* | China |
| **95** | **KT186231** | *Trichuris* sp. | *Trachypithecus francoisi* | China |
| **96** | **KT186234** | *Trichuris* sp. | *Trachypithecus francoisi* | China |
| **97** | **KJ588133** | *Trichuris* sp. | *Homo sapiens* | Uganda |
|  | KJ588134 | *Trichuris* sp. | *Homo sapiens* | Uganda |
|  | JN181833 | *T. trichiura* | *Homo sapiens* | Uganda |
|  | JN181844 | *T. trichiura* | *Homo sapiens* | Uganda |
|  | MN447327 | *Trichuris* sp. | *Macaca mulatta* | N/A |
| **98** | JN181834 | *T. trichiura* | *Homo sapiens* | Uganda |
| **99** | **KT344832** | *Trichuris* sp. | *Rhinopithecus bieti* | China |
|  | MH390367 | *Trichuris* sp. | *Rhinopithecus bieti* | China |
| **100** | **KP336477** | *Trichuris* sp. | *Macaca fuscata* | Italy |
| **101** | **KP336467** | *Trichuris* sp. | *Macaca fuscata* | Italy |
| **102** | **KT344829** | *Trichuris* sp. | *Macaca mulatta* | China |
|  | MH390367 | *Trichuris* sp. | *Macaca mulatta* | China |
|  | MH390368 | *Trichuris* sp. | *Macaca mulatta* | China |
|  | MH390369 | *Trichuris* sp. | *Macaca mulatta* | China |
| **103** | **KP336476** | *Trichuris* sp. | *Macaca fuscata* | Italy |
| **104** | **KP336474** | *Trichuris* sp. | *Macaca fuscata* | Italy |
|  | KP336473 | *Trichuris* sp. | *Macaca fuscata* | Italy |
| **105** | **KP336475** | *Trichuris* sp. | *Macaca fuscata* | Italy |
| **106** | **KP336464** | *Trichuris* sp. | *Macaca fuscata* | Italy |
|  | AB586133 | *T. trichiura* | *Macaca fuscata* | Japan |
| **107** | **KP336463** | *Trichuris* sp. | *Macaca fuscata* | Italy |
|  | KP336462 | *Trichuris* sp. | *Macaca fuscata* | Italy |
|  | KP336461 | *Trichuris* sp. | *Macaca fuscata* | Italy |
|  | KP336460 | *Trichuris* sp. | *Macaca fuscata* | Italy |
| **108** | **KP336468** | *Trichuris* sp. | *Macaca fuscata* | Italy |
|  | KP336465 | *Trichuris* sp. | *Macaca fuscata* | Italy |
|  | KP336466 | *Trichuris* sp. | *Macaca fuscata* | Italy |
|  | KP336469 | *Trichuris* sp. | *Macaca fuscata* | Italy |
|  | KP336470 | *Trichuris* sp. | *Macaca fuscata* | Italy |
|  | KP336472 | *Trichuris* sp. | *Macaca fuscata* | Italy |
| **109** | **KP336471** | *Trichuris* sp. | *Macaca fuscata* | Italy |
|  | KP336459 | *Trichuris* sp. | *Macaca fuscata* | Italy |
| **110** | **KJ588167** | *T. trichiura* | *Colobus guereza* | Uganda |

Our sequences:

| **9965_GOR_CAM** | **MW528545** | *Trichuris* sp. | *Gorilla gorilla* | Cameroon |
| --- | --- | --- | --- | --- |
| **771_CHI_CAM** | **MW528546** | *Trichuris* sp. | *Pan troglodytes* | Cameroon |
| **4880_CRP_CAM** | **MW528547** | *Trichuris* sp. | *Cercopithecus nictitans* | Cameroon |
